# A Functional Atlas of the Cerebellum Based on NeuroSynth Task Coordinates

**DOI:** 10.1007/s12311-023-01596-4

**Published:** 2023-08-22

**Authors:** Frank Van Overwalle, Qianying Ma, Naem Haihambo, Tom Bylemans, Beatriz Catoira, Mahyar Firouzi, Meijia Li, Min Pu, Elien Heleven, Chris Baeken, Kris Baetens, Natacha Deroost

**Affiliations:** 1 Faculty of Psychology and Center for Neuroscience, Vrije Universiteit Brussel, Pleinlaan 2, 1050 Brussels, Belgium; 2 Department of Psychiatry, Universitair Ziekenhuis Brussel, Brussels, Belgium; 3 Department of Psychiatry, Ghent Experimental Psychiatry Lab, Ghent University, Ghent, Belgium

**Keywords:** Functional atlas, Parcellation, Cerebellum, Neural networks, Ontological terms

## Abstract

Although the human cerebellum has a surface that is about 80% of that of the cerebral cortex and has about four times as many neurons, its functional organization is still very much uncharted. Despite recent attempts to provide resting-state and task-based parcellations of the cerebellum, these two approaches lead to large discrepancies. This article describes a comprehensive task-based functional parcellation of the human cerebellum based on a large-scale functional database, NeuroSynth, involving an unprecedented diversity of tasks, which were reliably associated with ontological key terms referring to psychological functions. Involving over 44,500 participants from this database, we present a parcellation that exhibits replicability with earlier resting-state parcellations across cerebellar and neocortical structures. The functional parcellation of the cerebellum confirms the major networks revealed in prior work, including *sensorimotor*, *directed (dorsal) attention*, *divided (ventral) attention*, *executive control*, *mentalizing (default mode)* networks, tiny patches of a *limbic* network, and also a unilateral *language* network (but not the *visual* network), and the association of these networks with underlying ontological key terms confirms their major functionality. The networks are revealed at locations that are roughly similar to prior resting-state cerebellar parcellations, although they are less symmetric and more fragmented across the two hemispheres. This functional parcellation of the human cerebellum and associated key terms can provide a useful guide in designing studies to test specific functional hypotheses and provide a reference for interpreting the results.

**Supplementary Information:**

The online version contains supplementary material available at 10.1007/s12311-023-01596-4.

## Introduction

The cerebellum takes up a large volume of the human brain, which has expanded more rapidly than the neocortex during evolution of humans and other great apes [5]. The cerebellum has a surface that is about 80% of the size of the cerebral cortex [6] and has about 4 times as many neurons [7]. There are strong structural and functional interactions between the cerebellum and cerebral cortex, with closed-loop connections running from the cortex to the cerebellum and back to the same areas in the cortex [8, 9]. This intrinsic connectivity has led to the discovery that the cerebellum and cerebral cortex share major functional networks underlying a variety of human psychological processes [1]. This important finding ran parallel to a change in research focus on the cerebellum—from a selective interest in motor processes to a growing awareness that the human cerebellum supports major cognitive, affective, and social functions ([1, 10–19].

What does the cerebellum contribute to these distinct functions? Research during the last decades has uncovered distinct functional neuronal networks in the brain using resting-state functional connectivity of functional imaging data, exploiting the observation that spontaneous fluctuations of activation signals can give insight in the intrinsic functional network organization of the brain. Based on this principle, seminal parcellations of the cerebrum [4] and the cerebellum [1], based on resting-state data of 1000 participants, have uncovered a solution of 7 shared network structures (and also a 17-network structure; for details see [4], and [1]). These encompass the *sensorimotor*, *visual*, *limbic*, *dorsal (directed) attention*, *ventral (divided) attention*, *executive control*, and *default mode* networks. These networks were largely replicated by Ji et al. [2] and were remarkably robust and valuable in elucidating functional brain organization in both the cerebral cortex and cerebellum, and provide a framework for functional interpretation of fMRI studies that is still useful today.

It has been argued that resting-state connectivity on which brain parcellations were initially based may reveal the pathways over which cognitive task activations flow [20, 21]. However, this is a largely untested assumption for the cerebellum. Defining or identifying the psychological processes subserved by these major network structures in the cerebellum is still a challenging issue, and it raises the question whether the major network structures identified in resting-state cerebellar parcellations reflect distinct functional processes. Therefore, establishing an atlas of distinct task-related processes in the cerebellum based on functional MRI (fMRI) activations is an important focus. In this study, we will provide a novel functional cerebellar atlas using task-based imaging data and explore its convergence with earlier task-free resting-state parcellations of both the cerebellum [1, 2] and cerebral cortex [2, 4] to validate the functionality of these earlier parcellations of the cerebellum.

Prior attempts using task-based imaging data to unravel the functionality of the human cerebellum were limited in several respects. First, prior studies on the task-based organization of the human cerebellum were based on meta-analyses using a limited set of task domains [13, 16, 17], which limits the generality of these findings with respect to an overall functional structure of the cerebellum. Fortunately, more recent studies included a larger range of task domains. One of the earliest major attempts to uncover a task-based functional parcellation of the whole cerebellum was conducted by King and colleagues [3] and involved 24 participants. King et al. [3] based their parcellation on 26 unique tasks to characterize motor and cognitive processes, including high-level cognition such as language and math. However, this task selection has limitations. For example, among the many cognitive tasks, they included only one social task for identifying the capacity to *mentalize*, that is, understanding the mind of another person, also known as *theory of mind* [22–24]. However, recent meta-analyses have robustly demonstrated that social mentalizing recruits a major part of the posterior human cerebellum [18, 19, 25–27]. Complicating matters further, although a surface overlap may appear between *mentalizing* and *language* at the group level, these functions shown little overlap in individual participants and should therefore be distinguished ([28]; see also [2]). Hence, prior cerebellar parcellations (e.g., [3]) may have been biased, leading to an underrepresentation of important functions such as social cognition and an overrepresentation of other functions such as language.

Second and perhaps most importantly, the identification of the psychological processes underlying task-based parcellations of the human cerebellum were not guided by strong and independent empirical evidence. Earlier cerebellar parcellations focused on single predefined functional networks (e.g., social cognition: [25]), areas, and populations (e.g., temporoparietal junction and autism: [29]), which limits the generality of the findings. In a recent attempt to overcome these limitations, King et al. [3] defined the psychological processes underlying their task-based cerebellar parcellation by applying a post hoc and independent cognitive ontology [30], but the resulting descriptors included distinct and diverse processes for the same regions (e.g., divided attention and verbal fluency describing region 6). This approach led to limited convergence between their task-based cerebellar parcellation and other task-free parcellations ([1, 2]; see also Fig. 4). The lack of a more unequivocal functional nomenclature in recent work renders interpretations of the discovered cerebellar structures ambiguous and insight in the functionality of the cerebellum difficult.

To address these limitations of earlier parcellations of the human cerebellum, we first identify a parcellation of functionally homogenous clusters based on a very large, publicly available database of past fMRI research, NeuroSynth [31], which showed convergent validity with earlier clusters. Second, we assign interpretable psychological concepts to these clusters. While the first aim is very similar to earlier attempts at parcellation (but relying on a larger database), the second aim is different and attempts at improving earlier analyses of cerebellar functionality.

Indeed, an important advantage of NeuroSynth is that psychological functionality is characterized by topical key terms extracted from the text corpuses of the articles in the database using latent Dirichlet allocation (for more details, see [32]). Topic mapping was then used in NeuroSynth to identify the relationship between these key terms and brain activation to characterize basic operations that reflect latent underlying mental functions that give rise to associated patterns of brain activity. This provides NeuroSynth with a strong empirical basis for conceptual functional description. To take advantage of this functional database, we (a) first selected pre-identified NeuroSynth topical key terms reflecting all unique task functions, (b) then performed a cluster analysis on all studies associated with each of these terms in NeuroSynth, and (c) finally labeled the cerebellar surface using a winner-take-all approach of the obtained clusters and their associated task functions. It should be noted, however, that although our approach rests on NeuroSynth’s convergence between researchers’ thinking and vocabulary about fundamental psychological processes, this convergence may well reflect systematic preconceptions and biases, which are only proxies for actual processes and may eventually turn out to be largely incorrect to characterize the functionality subserved by the cerebellum.

We are aware of several other biases of the NeuroSynth database, which in our opinion do not outweigh its advantages. First, some research topics in the database might have been published more or less frequently than others, and the quality of research might differ between research topics, which may have biased the data. Second, the NeuroSynth database is agnostic to imaging parameters and other analytic choices in the studies (e.g., spatial normalization and significance thresholds based on peak voxels or clusters), resulting in large variations with respect to the spatial localization and strength of activity. Third, the database provides only a summary of activation data reporting only peak coordinates, without any measure of inter-participant variability. However, we suspect that the sheer breadth and variety of research designs, tasks, and topics largely protect against these first three limitations, or, at least, are not more damaging than in the majority of empirical reviews and meta-analyses in the neuroimaging literature which suffer from these same limitations. Moreover, the number of participants included—over 40,000 for studies involving the cerebellum—is far beyond what has been analyzed so far, and hence allows to generalize across individual differences in functional organization.

Fourth, the cerebellum is incompletely covered in many studies, often leaving out the inferior cerebellum or having it preprocessed and analyzed inappropriately [33]. Coordinates of the inferior cerebellum are reported in far less studies than the superior cerebellum. (This can be verified in the activation maps in Supplementary Figure S1, where the inferior parts of the cerebellum show less signal). However, our winner-takes-all approach largely protects against this bias, because it compares activation of different clusters at voxel-level, that is, within the same spatial location (e.g., for each given voxel, a task with the highest activation will win the competition from other tasks with lower activation; if all activations for all tasks are much weaker in the inferior part of the cerebellum to the same degree, say only 50%, this same task will win the competition again in the inferior cerebellum). It is unlikely that a systematic and biased association would exist between neglect of the cerebellum and some task domains. Fifth, more generally, the cerebellar cortex is much more folded than the cerebral neocortex, so that localization of functional divisions might be more challenging (i.e., adjacent voxels only 1 mm apart could actually be located at different sides of a cerebellar sulcus and represent entirely different functions). Sixth, NeuroSynth data were acquired automatically, including much more than just fMRI activation such as connectivity, anatomical, and volumetric analyses. Hence, before using this database for analyzing the organization of the human cerebellum, a manual clean-up of the data was mandatory.

To sum up, this study attempts to identify a cerebellar parcellation with the aim to investigate spatial convergence with major functional networks uncovered by earlier resting-state analyses [2, 3, 34] and earlier task-based approaches such as King et al. [3]. However, we take a novel approach by strongly relying on the mental functions of brain structures empirically identified by the task-related brain database of NeuroSynth [31, 32] to define the psychological processes associated with our cerebellar parcellation.

## Method

In brief, our analysis took the following steps roughly similar to King et al. [3], which are explained in more detail below (see Supplementary Material for a technical summary):

1. Selection of studies NeuroSynth database with MNI coordinates in the cerebellum
2. Selection of functional NeuroSynth topics and terms from the database *v5-topics-50*
3. Quality screening of selected studies and coordinate tables
4. Meta-analytic processing of the selected functional topics using the activation likelihood estimation (ALE) procedure
5. Assigning clusters to anatomical areas on the z-values obtained by ALE
6. Representing the clusters on the cerebellar surface based on a winner-takes-all approach

### Selection of Cerebellar Coordinates

Coordinates were identified as cerebellar, using the following inclusion criteria (all of which had to apply):

1. When coordinates were provided in MNI coordinates, since the automatic conversion of other coordinate systems into the MNI system is often flawed in NeuroSynth (see also [18])
2. When coordinates were located within an anatomical cerebellar mask loosely created around the cerebellum, using several anatomical restrictions:

- A box defined by the outer x-y-z coordinates −63 > x < 63; -28 > y >−103; and z < 11
- Below z coordinates surrounding the superior part of the cerebellum determined by two planes defined by x-y-z coordinates 0 −50 12 and ±60 −50 −30, using the formulas: z < (−30 −12) / (60 − 0) * |x| +12; and z < −0.70 * |x| +12
- Below z coordinates surrounding the posterior part of the cerebellum determined by x-y-z coordinates: 0 −50 12 and 0 −100 −15, using the formulas: z < (−15 −12) / (−100 + 50) * (y +50) + 12; and z < 0.54 * (y +50) +12

3. When coordinates received at least one cerebellar label by one of the following atlases (to be as inclusive as possible):

- Using the SPM Anatomy toolbox, the label “cerebellum,” “cerebellar,” “vermis,” “dentate,” or “lobule”
- Using the Talairach Client toolbox and after conversion to Talairach coordinates (cf. [35]), the label “cerebellum”

### Selection of Functional NeuroSynth Topics and Terms

We selected several central key terms from the NeuroSynth topics in the Database v5-topics-50. This database contains a set of 50 topics created by applying a standard topic modeling approach [32] to the abstracts or text of articles in the database, leading to a list of key terms that characterize each topic. The v5-topics-50 terms were extracted from the abstracts of all articles in the NeuroSynth database as of July 2018 (14,371 articles). Importantly for the present analysis, all the articles in the database are characterized by a loading on each key term (between 0 and 1). For each topic, the key terms are ordered along the highest loading to the topic, which allows to identify the most related key terms and articles. However, to extract the most central and unique key terms for each topic, we introduced additional criteria for the present analysis as follows:

1. We included 10 functional terms with the highest 10 loadings on each NeuroSynth topic.
2. We excluded all non-functional terms referring to areas of the brain, methodology of brain research (volumes, network, state, resting, …), methodology of stimulus presentation (e.g., priming, response, …), and clinical pathologies and populations (e.g., neuroticism, bipolar, ms, ad, …). After eliminating these non-functional top 10 terms, the total list of 50 topics was reduced to a total of 22 functional topics.
3. From the remaining top 10 terms, we selected a maximum of five key terms that were unique across the 22 remaining topics, so that they were representative for that topic only (see Table 1 “Selected functional key terms”); in addition, closely related terms from the top 10 terms were added (i.e., with the same word stem, e.g., “trait” and “traits”).
4. To ensure that the selected terms (and especially the first term) were central to the topic, we included only terms with sufficiently low within-topic uniqueness and high within-topic convergence with respect to the first, most representative term of the topic.

- *Uniqueness* within a topic was calculated by the relative increase of studies in the NeuroSynth database after adding an additional term to the first (i.e., most representative) term using the formula: #(1 to a) / #(1 to [a − 1]), where # reflects the number of studies including the first term and all additional terms “a” up to that point divided by the number of studies up to the previous point “a – 1.” To illustrate, for the addition of the third term, the uniqueness formula would become: #(1 to 3) / #(1 to 2).
- *Overlap* within a topic was calculated by the relative overlap of studies in the NeuroSynth database after adding the term using the formula: (#1∩#a) / #(1 to a) where # reflects the number of studies of the first term and of the additional terms “a” divided by the number of studies including the first term and all additional terms “a” up to that point (as defined above). To illustrate, for the addition of the third term, the formula would be: (#1∩#3) / #(1 to 3).

**Table 1:** Selected functional key terms from the NeuroSynth database in decreasing order of centrality for each topic, and ALE parameters

NeuroSynth topic	Selected functional key terms					Centrality key terms		ALE analyses			
	Term 1	Term 2	Term 3	Term 4	Term 5	Mean convergence	Mean uniqueness	Cerebellar coordinates	Whole brain coordinates	Studies	Participants
1	trait	traits	personality			29	68	531	3023	86	2624
7	reward	rewards	feedback	anticipation		53	87	1353	8823	236	6293
8	mind	tom				94	25	378	2560	54	1164
9	working	wm	load			68	55	1372	7637	184	4519
11	training	trained	practice	sequence		33	98	1647	7760	183	4111
13	fear	threat				31	36	706	2819	79	2099
16	inhibition	inhibitory	stop			55	59	862	4554	113	3461
17	sensorimotor	finger				23	34	1438	7946	156	3104
18	number	numerical	arithmetic	magnitude		33	88	1527	8380	197	5086
19	action	actions	observation	mirror		47	88	1962	13,145	308	6340
20	conflict	interference	incongruent			22	77	890	5981	142	3504
25	location	space				15	38	668	4193	111	2108
26	emotion	affective	valence			33	65	1696	10,852	281	7547
28	empathy	empathic	moral			39	63	396	3215	66	1634
30	decision	decisions	choice	choices	risky	59	110	992	6326	197	4566
31	prediction	predictive	predictions	classification		18	105	1086	6352	162	3796
33	encoding	episodic	retrieval			33	68	2493	14,761	338	9798
37	word	sentences	sentence	reading		32	89	1765	11,182	290	6395
38	objects	category				17	35	1211	7037	208	4659
40	face	faces	facial	expressions		44	90	1817	9090	247	6294
41	events	scenes	scene			10	93	1489	7891	188	4302
47	attention	attentional				57	30	1620	10,440	293	6542
Totals (ignoring contributions to multiple topics)								12,105	71,290	1820	44,506

Functional key terms were ordered along decreasing centrality from terms 1 to 5. Centrality of a key term for a topic is indicated by high overlap of studies sharing the same key term and a low number of studies with unique additional key terms related to the topic but not sharing that key term. This was computed (on all NeuroSynth studies) for two measures with respect to the most representative term 1: Mean overlap = % of studies with key terms 2–5 also sharing key term 1 (averaged across key terms 2–5). Mean uniqueness = % of studies with unique key terms 2–5 not sharing key term 1 (averaged across key terms 2–5). These measures followed the order of terms in NeuroSynth, except in a few cases were key terms were reordered or omitted based on these centrality measures. Centrality measures were conducted on studies before the Quality Check. Totals ignore repetitions due to multiple contributions of some studies to more than one topic. *tom*, theory of mind; *wm*, working memory

Based on this analysis, 5 key terms were omitted with an overlap under .09 (i.e., at 25% of the mean overlap across all topics), while some key terms were reordered (i.e., put earlier) because these terms had relatively higher centrality scores (i.e., higher overlap and lower uniqueness)—this reordering impacts only the use of terms in the results, tables, and figures, but not the selection of studies. Table 1 shows the final selection of the key functional terms for each of the 22 selected functional NeuroSynth topics.

### Quality Screening of Selected Studies and Coordinate Tables

We included studies that investigated task-related brain activation using fMRI. This was done through inspection of each of the original articles by many co-authors (in alphabetical order: Kris Baetens, Tom Bylemans, Beatriz Puerta Catoira, Mahyar Firouzi, Naem Haihambo, Meijia Li, Qianying Ma, Min Pu, and honor students), using the exclusion and inclusion criteria listed below. The raters read minimally the abstract of each article, and in case of inclusion, the full article insofar as to obtain necessary information and check the criteria. The decisions of the raters were double checked by Qianying Ma and Naem Haihambo, and disagreements were resolved by discussion:

1. *Excluding* studies or tables referring to analyses with (in alphabetical order) connectivity or PPI, coordinates that were incorrect or from individual participants, coordinate tables that were not found, meta-analyses, neuromodulation, neurostimulation, patients (excludes whole study), PET, resting-state, SPECT, substance, or drug use (excludes whole study), volume, young participants (< 12 years) (excludes whole study). We did not distinguish between activations or deactivations, or the direction of contrast in the analyses.
2. *Including* studies or tables referring to analyses with (in alphabetical order) covariate analysis, multivariate analysis, parametric analysis, regression analysis, SUIT analysis or space, and other analyses that reflect task-related brain activation.

Information on the number of participants was added into the analysis. In case of multiple experiments reported in a single table, we entered the total number of all participants of the table/study for a joint analysis, or the average of all participants of the table/study for separate analyses.

### Functional Parcellation

This analysis proceeded in the following steps (using custom-made Matlab scripts, except in step 1). All calculations are based on the volume of the cerebellum (and cerebrum), except when noted otherwise:

1. ALE analysis on the 22 functional topics. Activation likelihood estimation (ALE) analyses were conducted on the coordinates of all studies related to the selected key terms for each of the 22 functional topics, as implemented by GingerALE 3.0.2 [36–39]. ALE reveals the consistent activation across studies for each topic and results in a map of unthresholded z-values. No clusters or thresholds were defined as the purpose of further analysis was to look for the highest z-value in each voxel for each topic to determine the winner-take-all dominant functionality. Table 1 lists the number of coordinates, studies, and participants involved for the 22 functional topics, and Supplementary Figure S1 depicts the z-value flatmaps [40].
2. Clustering of functional topics. We applied hierarchical clustering on the ALE analyses of the 22 functional topics, after rescaling all z-scores for each topic between 0 and 1 (i.e., unity-based normalization) to minimize activation differences related to the experimental tasks that are characteristic for each topic. Normalization is often applied in resting-state parcellation across participants [1, 2], while we apply it here across ALE topics in order to obtain equivalent results. It logically tends to favor ALE topics that have overall lower activation (see Supplementary Figure S2 for a visual comparison, with or without normalization, after applying step 3). Hierarchical clustering allows to decide on the number of clusters based on the existing pattern or dendogram of hierarchically nested topics in clusters that are meaningful, rather than setting a number of clusters a priori. We used hierarchical clustering methods that yield compact and relatively independent clusters, including Ward [41] and Complete linkage [42] based on Euclidean distance between the cerebellar coordinates, using the *clusterdata* procedure of Matlab 2021.
3. Assigning clusters to anatomical areas. We decomposed the T (topics) × V (voxels) z-value matrix into a product of an T × C (cluster) matrix of task profiles and a C × V matrix of voxel weights. A winner-takes-all approach was adopted to assign each voxel to the cluster with the highest z-value. To smooth the pictorial representation of the parcellation, the parcellation was filtered using a box of isometric voxels surrounding each voxel and assigning each voxel to the most frequent cluster in the box after 10 random sweeps through the data.
4. Spatial homogeneity of the parcellations. To measure whether the parcellations were spatially homogenous, we calculated the distance-controlled boundary coefficient (DCBC) introduced by King et al. [3]. The basic idea is that if a boundary divides two parcellations, then any equidistant pair of voxels within a parcellation should have activation profiles that are more correlated with each other than two voxels that are separated by the boundary. Note that this assumption creates a bias as it favors parcellations with smooth closed curvatures (e.g., circle) over other shapes (e.g., banana-like shapes), and favors clear lines at boundaries over small irregularities such as wrinkles or fragmented patches (intruding from the other parcellation). Relatedly, given the extreme folding of the cerebellar cortex, adjacent voxels only 1 mm apart could actually be located at different sides of a cerebellar sulcus and hence in different networks. As these spatial shapes of boundaries are not well controlled for, the DCBC should be interpreted with some caution. We calculated correlations between voxel pairs using a range of spatial bins (i.e., with Euclidian distance of 6 mm, 10 mm, and so on up to 50 mm; with each distance rounded to 2 mm of the minimal voxel size). The difference between the within- and between-parcellation correlations for each spatial bin then serves as the DCBC. We followed the implementation of King et al. [3], and calculated Pearson correlations of the final parcellation (without truncating negative covariances, as we found no strong motivation for it).
5. Similarity between parcellations. To compare the similarity between different parcellations from prior and present analyses, we resampled all data sets in a common SUIT data format (using suit_map2surf), and calculated a Rand similarity index (which ranges between 0 and 1), as well as the adjusted Rand index reflecting similarity above chance level (when the value is above 0; [43, 44]; see code by [45]). We report both indices because of the controversy involving the adjusted index as it depends on the chosen random distribution [46] and is not entirely independent of the number and size of clusters either [44]. For a discussion of the results, we mainly focus on Rand indices with an adjusted value > 0. The target of comparison was restricted to the 7 networks, which were consistently found in Buckner et al. [1] and Ji et al. [2], and the additional language network of Ji et al. [2]. Consequently, clusters that were merged in single parcellations/networks in the present analyses were also merged for the computation of the Rand indices, while all remaining clusters were collapsed in a single “other” category. To compare the similarity among individual networks, we conducted the same analysis for each network individually, while the other networks not involved were assigned to a single “other” category.
6. Cross-validation of the functional parcellation. To obtain a measure of cluster reliability, we randomly divided the studies for each of the 22 topics in two non-overlapping halves, and computed a new ALE analysis on the two halves for each of the topics. We then derived a winner-take-all parcellation based on the same pre-determined functional clusters obtained in the previous step 3. Cluster reliability was determined by the Rand index between the winner-take-all results of the two halves. We repeated this cross-validation process 20 times, and averaged the Rand index. Note that this provides a lower bound on the reliability of the full parcellation, because the analysis is conducted on only half of the full data set.
7. Cross-validation through functional connectivity with the neocortex. To obtain convergent validity for the cerebellar parcellation, we conducted an analysis of the functional connectivity with the cerebral cortex. This essentially involved replicating step 3, now applied on the whole brain database of cerebellar studies, that is, including all coordinates of the entire brain (cerebral and cerebellar structures) of the studies with cerebellar coordinates.

## Results and Discussion

To briefly recapitulate, our goal was to conduct a task-based parcellation of the cerebellum, using a much larger dataset than has been used so far. To do so, we selected studies from the NeuroSynth 50 topics database from 2018 [31], which reported cerebellar involvement (using an approximate cerebellar mask and the SPM Anatomy and Talairach Client toolboxes) and activation in MNI coordinates. The database involves 50 topics represented by key terms describing basic operations that reflect latent underlying mental functions giving rise to associated patterns of brain activity [32]. We included 22 topics that indicated functional task-related processes. Each selected topic was denoted by a representative top term and up to four additional key terms from the database, all of which were unique to each topic to avoid functional overlap (Table 1). The top term had the highest load in the NeuroSynth database for that topic and a high centrality (i.e., high overlap and low uniqueness) with respect to the four additional key terms. For each topic, we extracted the MNI coordinates from the cerebellum as well as from the whole brain (cerebral and cerebellar structures, including only studies with cerebellar coordinates). We then conducted meta-analyses on all studies that were associated with each of the 22 functional topics, using the ALE procedure [36–39]. We conducted these ALE analyses on both the cerebellar data as well as on the whole brain data.

**Fig. 1:**
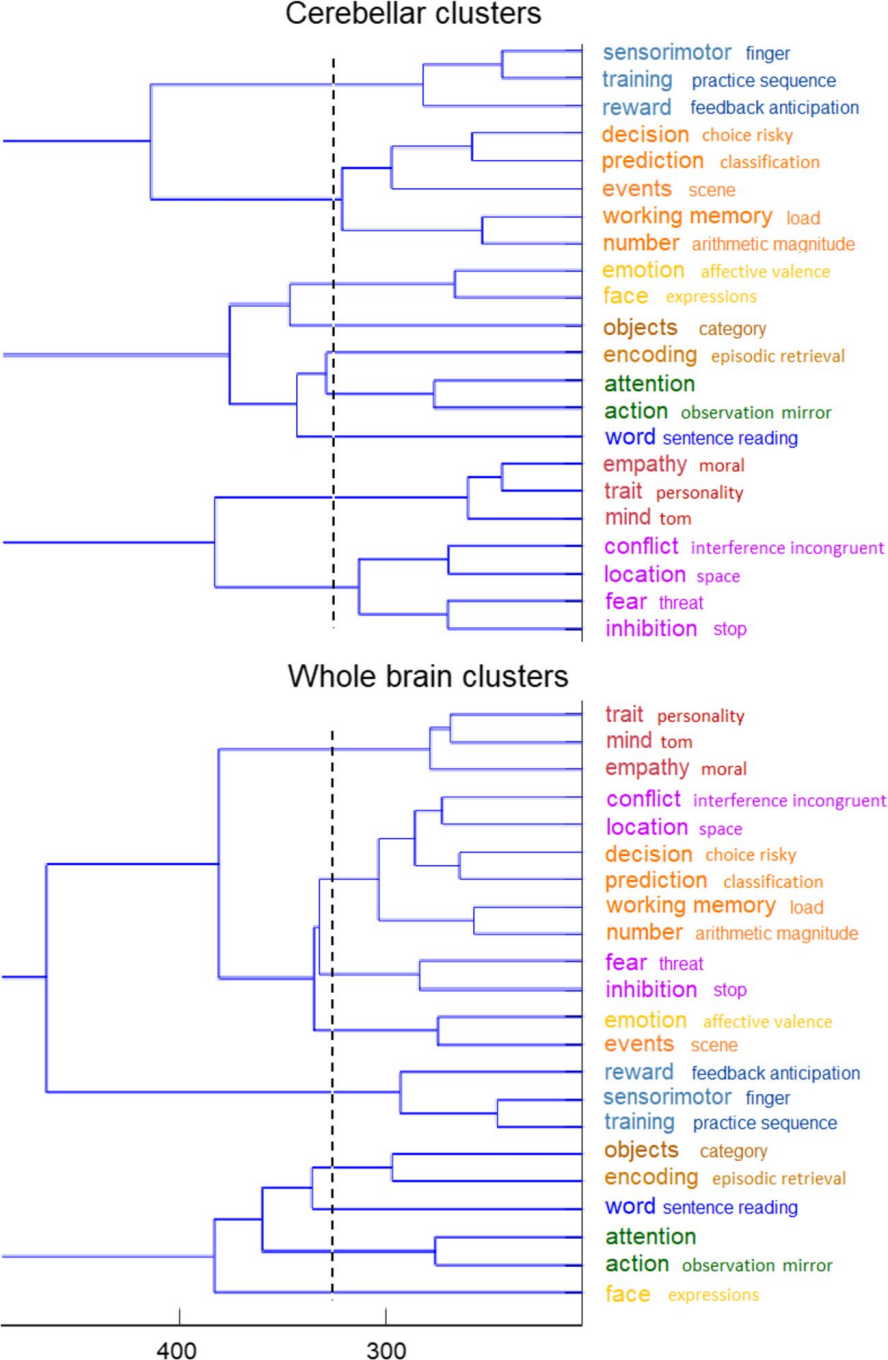
Functional cluster analysis of NeuroSynth activations. The analysis is based on the combined input of ALE z-scores on the 22 selected topics of the cerebellar and whole-brain (including cerebellar) MNI coordinates. The vertical broken line demarcates the solution for 9 clusters in each database, while the x-axis represents the distance between z-scores at which functional topics are clustered. By giving priority to cerebellar clusters (when equivalent whole-brain clusters were broken up), the analysis ended up with a final 10-cluster solution. To illustrate, the single “decision-prediction-events-working-number” cerebellar cluster yielded two whole brain clusters, which were relatively close to each other, and hence the single cluster was kept. Likewise for the single “conflict-location-fear-inhibition” cerebellar cluster. In contrast, the “emotion-face” cerebellar cluster yielded two very distant clusters in the whole brain, which were therefore separated. Colors refer to the spatial representation of the same clusters in Fig. 2

We then conducted a combined clustering on the Z values associated with the ALE analyses for both the cerebellum and whole brain data, using the hierarchical methods of Ward [41] and Complete linkage [42]. This combined analysis was driven by our attempt to seek convergence between cerebellar and cerebral networks, and so increase convergent validity of the clustering results. We arrived at a cluster solution for both the cerebellum and the whole brain, which was remarkably similar across the two brain parts and clustering methods. Although there were a number of equivalent ways for clustering, we selected the complete linkage clustering solution because of its somewhat superior overlap between the cerebellar and whole brain analyses, and also because it yielded more compact, and thus functionally more homogenous, clusters. The few remaining differences between the two brain parts were resolved by given priority to cerebellar clusters, if the distance on the whole-brain clusters were not too large. By doing so, we ended up with a 10-cluster solution (Fig. 1) in line with prior research arriving also at 10 clusters to describe the connectivity [2] and functionality [3] of the cerebellum. Using a winner-takes-all approach, we derived from this clustering a 10-cluster cerebellar parcellation.

### A 10-Cluster Parcellation of the Cerebellum

**Fig. 2:**
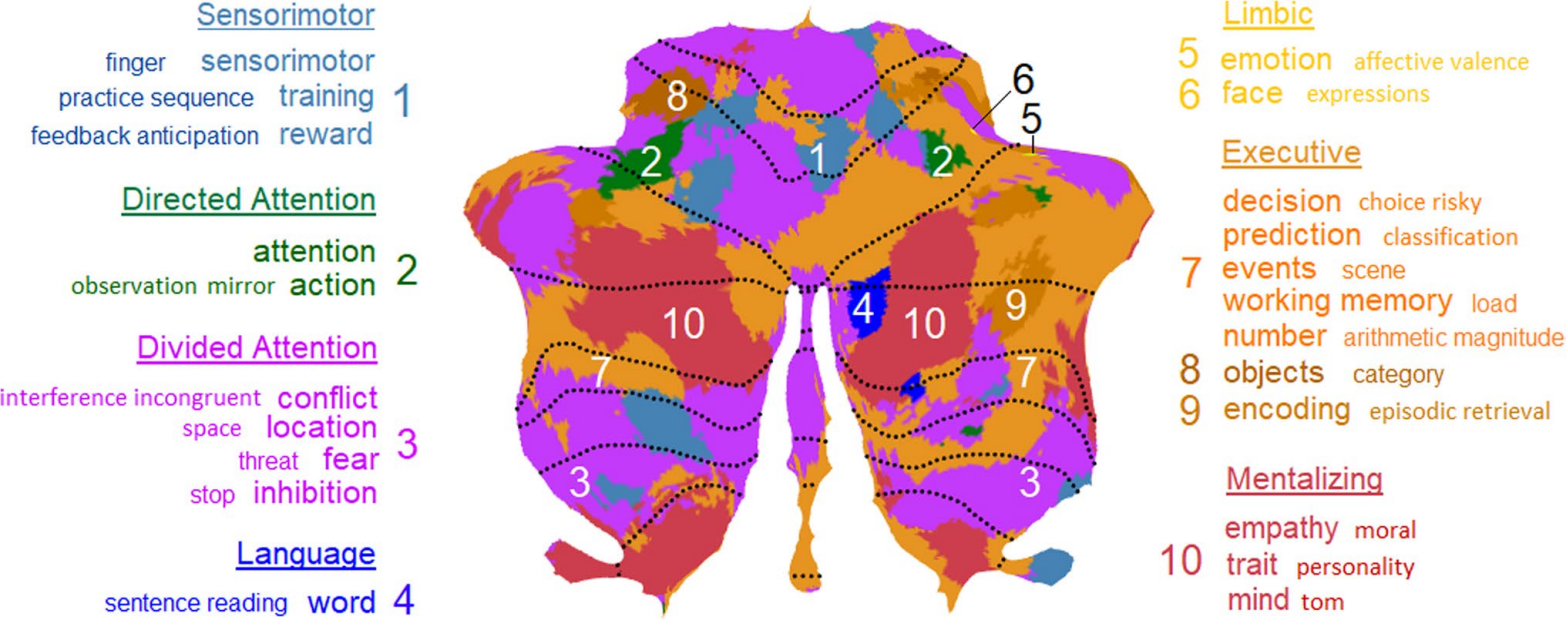
Parcellation of the cerebellum into 10 functional clusters. The identification of the “winning” clusters are based on normalized ALE z-scores and displayed on a cerebellar flatmap, smoothed with a 5 isometric voxels box filter. The top and additional key terms from NeuroSynth that characterize each “winning” cluster are listed with larger and smaller font respectively. The suggested major networks to which single or multiple clusters belong are also indicated, resulting in 7 networks overall. In the remainder of the text, the 10-network structure refers to the original 10 cluster solution here, while the 7-network structure refers to the 7 networks. Numbers for each cluster refer to the location on the flatmap. Colors refer to the same clusters as in Fig. 1. Note that the yellow color of the limbic clusters 5 and 6 is identical (given the tiny surfaces) and that the brown color of the executive clusters ranges from light brown (clusters 7), brown (cluster 9), to dark brown (cluster 8). Some colors in the legend are slightly darkened to improve readability. tom, theory of mind

Our cerebellar parcellation is represented on a cerebellar flatmap (Fig. 2; [40]). On a flatmap, the anterior parts of the superior cerebellar surface (lobule I) and inferior surface (lobules IX and X) are stretched and flattened from the top to the bottom respectively of the flatmap, with the most posterior parts of the cerebellum (lobule VII) in the middle. Thus, from top to bottom of the flatmap, the upper part reflects the anterior cerebellum (lobules I to V), and the middle to bottom part reflects the posterior cerebellum (lobules VI to X). An advantage of a flatmap is that the surface structure and volume of a parcellation is immediately visible. We describe the present results from top to bottom on the flatmap.

Starting at the superior surface, we clearly observe the bilateral *sensorimotor* network in the anterior cerebellum (cluster 1) although it is quite fragmented, a large but also fragmented *divided (ventral) attention* network (cluster 3), and two tiny areas representing the *limbic* network at the right hemisphere (clusters 5 and 6). This is followed by a small bilateral *directed (dorsal) attention* network (cluster 2) and a large bilateral *executive* network (composed of clusters 7–9), ending in the posterior cerebellum with a *mentalizing* network, which constitutes the larger part of the *default mode* network ([47]; cluster 10). Turning back along the inferior surface (i.e., further down the flatmap), we observe at the right hemisphere an independent *language* network (cluster 4) bordering along two sides the *mentalizing* / *default mode* network. We further find the same networks at both hemispheres as on the superior surface. This large-scale structural division confirms the primary and secondary representation of the major networks on the superior and inferior surface, respectively, of the cerebellum (cf. [1]).

**Fig. 3:**
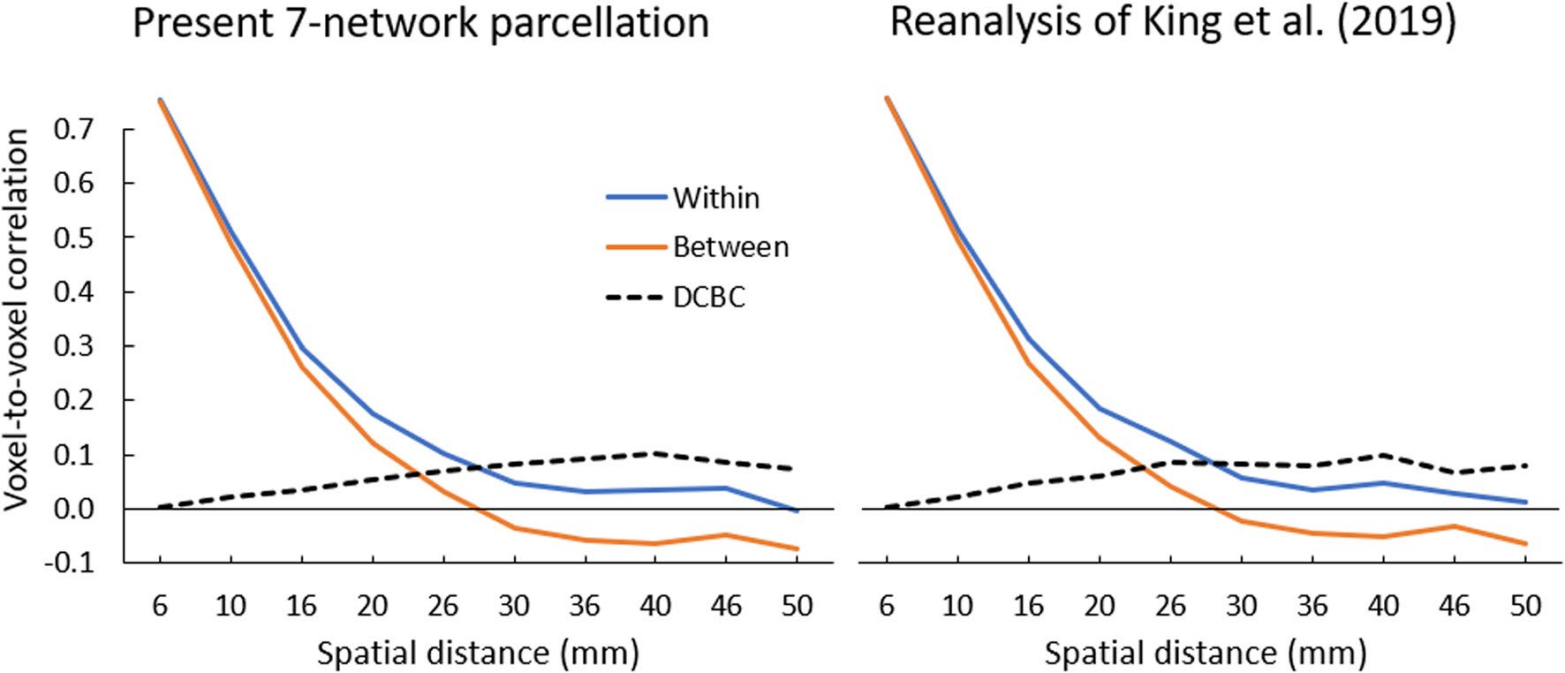
Spatial homogeneity of the parcellations. Correlations for “Within” (blue) and “Between” (orange) voxel pairs and resulting DCBC difference (dashed black) for [left] the present 7-network parcellation and [right] reanalysis of King et al. [3]. As can be seen also in Supplementary Table S2, the results of the two parcellations are very similar with equivalent DCBC results, suggesting a similar level of homogeneity

To evaluate the spatial homogeneity of the 10 cluster parcellation as well as of the derived 7-network parcellation (including a *language* network but excluding a *visual* network), we computed the DCBC [3], which reflects correlations between voxel pairs using a range of equidistance voxels (i.e., bins of the same spatial distance) and so effectively controls for spatial distance. The difference between the within- and between-parcellation correlations for each spatial bin is the DCBC index. For both the 10-cluster and 7-network parcellations, the strongest DCBC value was 0.10 at an equidistance of 40 mm (Fig. 3 left, Supplementary Table S2), demonstrating a spatial homogeneity of the parcellations, which was, however, lower than the DCBC of 0.16 reported in King et al. [3]. This statistical result probably reflects the more spatially fragmented clustering/networks of the present parcellation, but is unrelated to the functional validity of the clusters.

### A Comparison with Prior Task-Free and Task-Related Parcellations

**Fig. 4:**
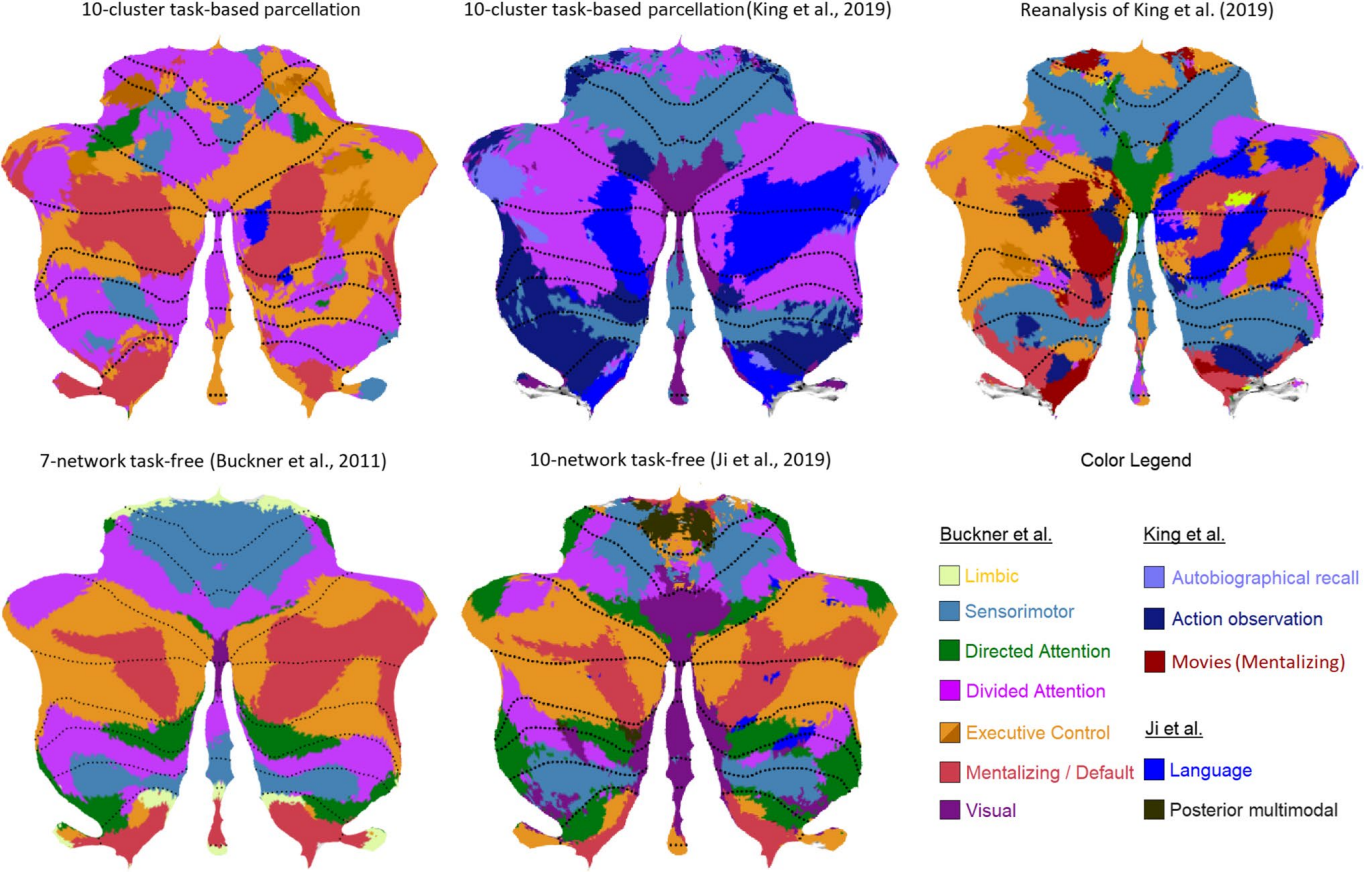
A comparison of cerebellar parcellations. [Top row] The task-based parcellation in the present study (top left), King et al. [3], and reanalysis of King et al. [3]; [bottom row] resting-state parcellations of Buckner et al. [1] and Ji et al. [2]; [bottom right] color coding legend applied to all parcellations for ease of comparison. The main color legend refers to the original color code by Buckner et al. [1] and is used for similar networks in all atlases (based on the original authors’ main characterization). Additional color codes from other authors refer to additional networks from other studies (see legend). The present task-based solution is most similar to the resting-state structure of Ji et al. [2] with respect to the networks included and their location (except for the visual and additional networks), and of Buckner et al. [1]

A visual comparison between our parcellation with the task-free parcellation by Buckner et al. [1] and Ji et al. [2], as well as the task-related parcellation of 10 networks by King et al. [3], is provided in Fig. 4. To facilitate this comparison, we used flatmaps and color-coded the same major functional networks along the Buckner et al. [1] 7-network structure. Unique networks identified by Ji et al. [2] and King et al. [3] are represented by additional color coding. Apart from the striking similarities of the present clustering solution with the network structures and locations of Buckner et al. [1] and Ji et al. [2], we can observe some important differences.

We begin with differences with Buckner et al. [1]: Our *limbic* network is only represented as a tiny cluster; our *sensorimotor* network is rather fragmented; and our *directed (dorsal) attention* network is bilaterally represented on the superior surface showing extensive areas, but is hardly discernable on the inferior surface. Interestingly, our parcellation reveals a clearly discernible *language* cluster on the right hemisphere that was not identified in the 7-network structure by Buckner et al. [1].

More similarities can be observed in the comparison with the parcellation by Ji et al. [2]. Almost like our 10-cluster structure, this 10-network parcellation fails to identify an independent *limbic* cluster. Another similarity is that their *sensorimotor* network is more fragmented than Buckner et al. [1]. Perhaps most importantly, Ji et al. [2] also revealed an independent *language* cluster mainly on the right hemisphere. Also noteworthy is that this parcellation identifies a larger network volume related to *directed (dorsal) attention* at the superior surface, much in line with our structure.

Similarities of the present analysis are also observed with the task-related 10-fold functional parcellation by King et al. [3], although there are many differences. We color-coded this parcellation in line with the top ontological term (i.e., with the highest feature weight) of each region (Fig. 4), in line with the winner-take-all approach of the other parcellations. At the superior surface, our parcellation does not reflect the large bilateral contribution of *sensorimotor* functions to *hand presses* and *motor planning* in King et al. [3]; see also [1]), and neither the representation of visual functions involving *saccades* and *visual processing* in and close to the vermis. In King et al. [3], there is no clear representation of an *executive*, *mentalizing* / *default mode*, or *limbic* network, except for *autobiographic recall*, which might be interpreted as an executive (i.e., recall) or mentalizing (i.e., autobiographic) function. King et al. [3] reveal a large representation of *divided attention* volumes at the left and right hemispheres, interrupted only by *narrative / language* processes closer to the vermis. This large *narrative / language* cluster strongly overlaps with the two distinct *mentalizing* and *language* clusters in our parcellation.

We computed the similarity between all the above parcellations using the Rand index, as well as the adjusted Rand index, which reflects convergence above random level when its value is above zero. As can be seen in upper panel of Table 2 (and focusing on Rand indices with adjusted values > 0), the results confirm the high similarity between the task-free parcellations by Buckner et al. [1] and Ji et al. [2] with Rand = 0.78, which is followed by a similarity of Rand = 0.75 of both task-free parcellations with the task-based parcellation by King et al. [3]. A somewhat lower (but still substantially better than random) similarity of Rand 0.66–0.67 is found between our 7-network task-based parcellation and earlier parcellations (both task-free parcellations and [3]).

**Table 2:** (Adjusted) Rand index comparing the similarity in parcellations of task-free and task-based studies

	Rand index			Adjusted Rand index		
	Buckner et al. [1]	Ji et al. [2]	King et al. [3]	Buckner et al. [1]	Ji et al. [2]	King et al. [3]
All Networks						
Ji et al. [2]	0.78			0.27		
King et al. [3]	0.75	0.75		0.23	0.19	
Reanalysis of King et al. [3]	0.75	0.73	0.75	0.22	0.14	0.26
Present 7-network parcellation	0.67	0.66	0.65	0.11	0.07	0.08
Means of all individual Networks						
Ji et al. [2]	0.80			0.25		
King et al. [3]	0.72	0.73		0.06	0.19	
Reanalysis of King et al. [3]	0.75	0.76	0.76	0.13	0.13	0.09
Present 7-network parcellation	0.74	0.75	0.73	0.10	0.10	0.02

This analysis is based on the 7 networks of Buckner et al. [1] and an additional language network of Ji et al. [2], and uses parcellations based on merged clusters in the last two studies (i.e., present analyses). All other clusters were collapsed in an “other” category. For individual network analyses, the networks not involved were also assigned to the “other” category

To analyze where the lower Rand indices from the present analysis may originate from, we also analyzed all individual networks separately (i.e., each of the 7 networks of [1], and the *language* network of [2]; see bottom panel of Table 2). This analysis showed relatively higher similarity with the two task-free parcellations for higher-level associative networks involving *executive control*, *default mode*, and *language*, while the King et al. [3] analysis showed higher similarity for the *sensorimotor* network (Supplementary Table S1). Overall, this resulted in somewhat better average convergence of the present work with the two task-free parcellations with mean Rand = 0.74–0.75, which is equivalent to King et al. [3] with mean Rand = 0.73. Note that the Rand index evaluates similarity between spatial locations and boundaries, but not the adequacy of ontological terms denoting the functionality of the parcellations.

Taken together, the comparison of earlier and our results clearly demonstrate important functional boundaries in the cerebellum. However, they also make clear that there are a number of closely equivalent ways to subdivide the cerebellum, which are constrained by the methods used for data sampling and clustering. Our parcellation provides clear similarities with the task-free structures identified by Buckner et al. [1] and Ji et al. [2], while the topological key terms provide a functional task-related characterization quite different from King et al. [3].

### Cross-Validation of the 7-Network and 10-Cluster Parcellation

To assess the consistency of our 7-network and 10-cluster parcellation, we conducted a cross-validation analysis using split-half reliability analysis. Specifically, for each of the 22 topics, we divided all studies randomly in two equivalent halves, and for each random draw, we computed a new ALE analysis for each of the topics. We then derived a novel parcellation based on the pre-determined functional clusters of the full analysis. We repeated this cross-validation process 20 times. Cluster consistency was determined by the average Rand index across the repetitions. Note that this split-half analysis provides a lower bound on actual consistency since it is based on only half of the data.

**Fig. 5:**
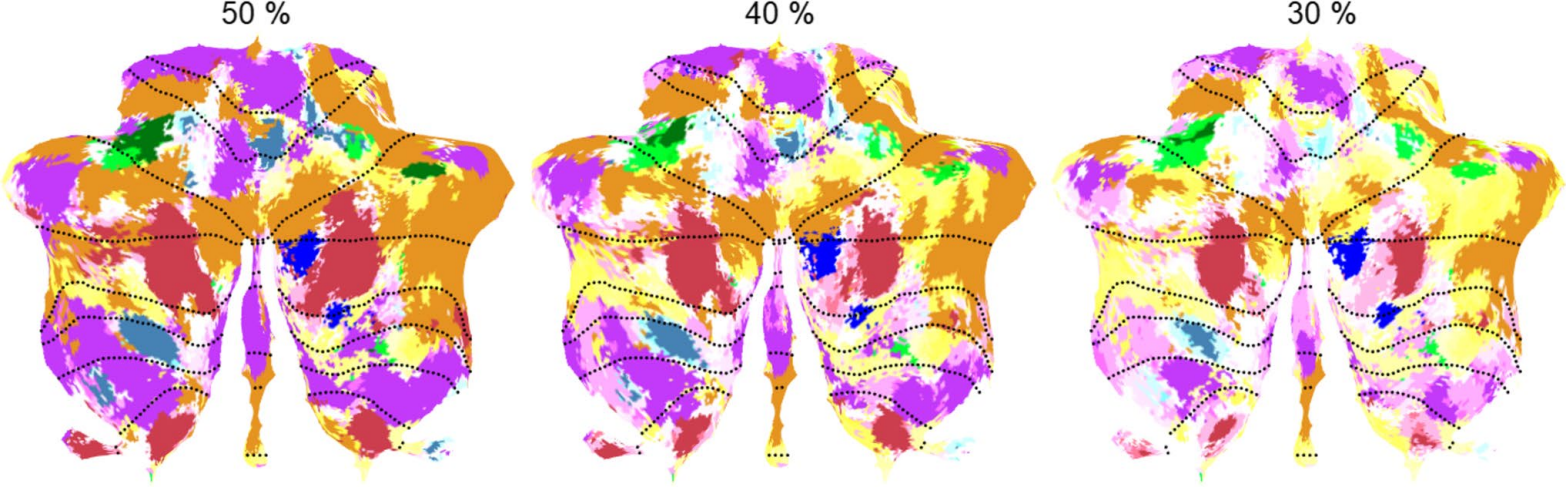
Visual illustration of split-halve reliability of the 7-network parcellation after 20 repetitions, with greater whitening reflecting less consistency among all split-halves. The illustration shows the original full network parcellation with whitening applied only when network displacement of voxels is beyond a threshold of 50 %, 40 %, and 30 % displacements across the repetitions. To illustrate, displacements that occur in more than 50% of the repetitions are less frequent than displacements in more than 30% of the repetitions, and this is shown in less whitening and more of the original structure for the 50% than 30% threshold

Overall, split-half reliability of the cluster structure was good with a mean Rand = 0.66 and 0.64 (adjusted Rand = 0.04 and 0.03) for the 10-cluster and 7-network parcellation, respectively, across 20 replications. This value is equivalent to the mean Rand = 0.65 of the cross-validation reported by King et al. [3]. When considering each network individually (with all other networks coding as belonging to an ‘other’ category), reliability increased with a mean Rand = 0.83 and 0.81, respectively (adjusted Rand = 0.55 and 0.48). There were no substantial differences between networks (see Supplementary Table S3). The consistency of the 7-network parcellation can be visually inspected in Fig. 5, where a whiter coloring indicates that voxels were assigned more often to a different network between the two random-split halves. As can be seen, the parcellation was most consistent for larger networks, including the *mentalizing*, *executive*, and *divided attention* networks, and also for the smaller *language* network.

### Validation Using a 10-Cluster Connectivity Parcellation of the Whole Brain

**Fig. 6:**
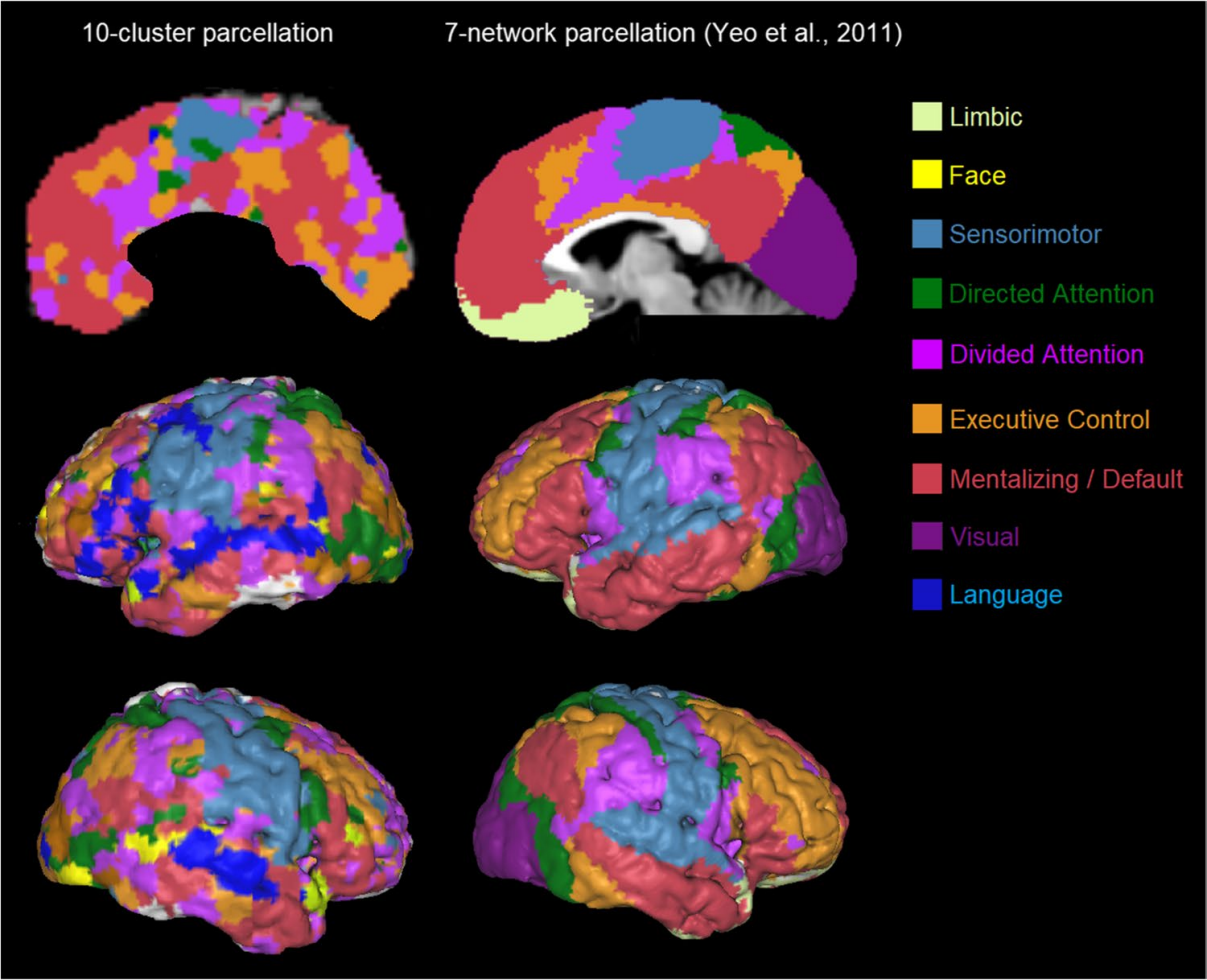
[Left] Functional connectivity between the cerebellum and the cerebral cortex. The “winning” clusters with the highest ALE *z*-values are displayed on the cerebral cortex. The clusters of the executive network are combined (brown color; as in Fig. 2). The networks are shown on a medial section and left and right hemispheres of the Colin brain using Mango, smoothed with a 3 isometric voxel box filter. [Right] The 7-network parcellation of the cerebral cortex by Yeo et al. [4]. Visual comparison shows that the present solution is close the 7-network solution by Yeo et al. [4], except that the present parcellation reveals a separate *language* network (since this was also identified in our cerebellar parcellation), and lacks a *visual* network (as it did not show up in our cerebellar parcellation) so that the occipital cortex is ‘erroneously’ substituted by other functions

To further validate our empirical approach on the parcellation of the cerebellum, we conducted a parcellation of the whole brain using the same 10 functional clusters, and analyzed both cerebellar and cerebral coordinates to establish coactivation between these two brain parts. As can be seen in Fig. 6, our 10-cluster whole-brain parcellation shows strong similarity with Yeo et al. [4] and other major networks structures identified also in other resting-state research (e.g., [2]), including *sensorimotor*, *directed (dorsal) attention*, *divided (ventral) attention*, *executive control*, *default mode*, and *limbic* networks, except for the *visual* network (see also Supplementary Figure S3). According to Buckner et al. [1], the primary visual network is not represented in the cerebellum, and consequently shows little convergence in the cerebral parcellation (on the occipital lobe). Importantly, the present parcellation also includes the bilateral language network identified by Ji et al. [2], which is somewhat smaller on the right hemisphere in the present and Ji’s parcellation. There is compelling similarity between the language network revealed here in the cerebrum (Fig. 6) and the core language structure identified in the literature ([48]; Fig. 1). Although differing at the edges of the networks, overall, our surface organization of the whole brain is remarkably robust and similar with earlier task-free parcellations. This result provides additional empirical evidence for our parcellation of the cerebellum. However, note that our initial cluster analysis rested on shared cerebellar and cerebral coordinates, and therefore this convergence cannot serve as an entirely independent validation of the present cerebellar parcellation.

### Validation Using a New Cluster-Analysis and Parcellation of King et al. [3]

**Fig. 7:**
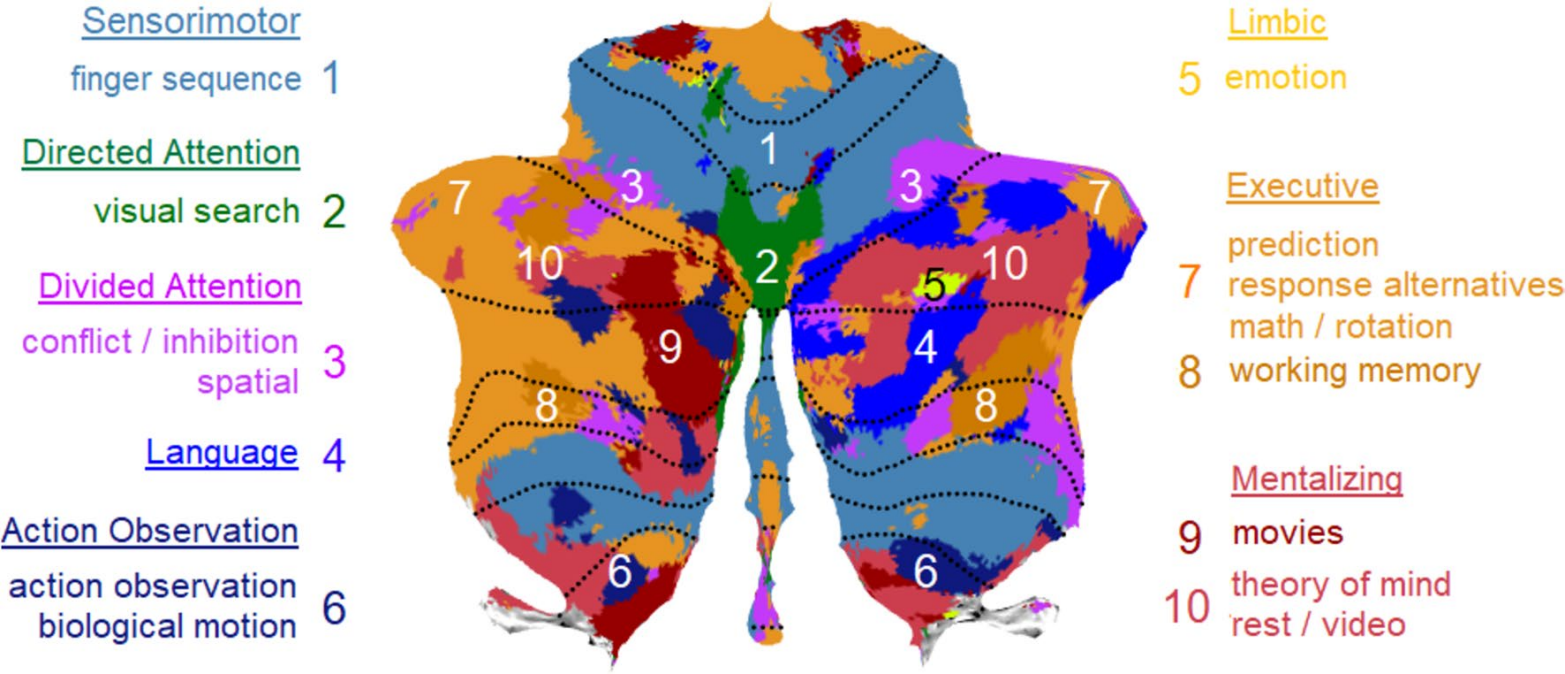
Functional clusters of the multidimensional task battery (MDTB) of King et al. [3]. The original 15 clusters were derived from the normalized task activation and are categorized into 10 major parcellations to display maximal similarity with the other cerebellar parcellations and network structures. The parcellation is displayed on a cerebellar flatmap, smoothed with a 5 isometric voxel box filter. The original cluster analysis of all tasks is shown in Supplementary Figure S4. Numbers refer to the location on the flatmap. For similar networks as in Fig. 2, identical colors (and numbers) are used; different colors refer to additional clusters (9: *movies*) or networks (6: *action observation*)

In another attempt to validate our empirical work, we sought convergence with the data of the multidimensional task battery (MDTB), which formed the basis of the King et al. [3] parcellation. We conducted a novel cluster-analysis and parcellation of the cerebellum on the 61 task activations of the MDTB, which are publicly available, using the same procedures as for our parcellation to answer the question: Is higher convergence possible when similar procedures are used? The reanalysis arrived at a 15-cluster solution using the hierarchical method of Ward [41], which maintained the best coherence between the same tasks across sets A and B and between conditions of the same task set. Figure 7 shows the results of the winner-takes-all approach, where we combined several neighboring clusters reflecting similar psychological processes under the same network, in the same way as we did for our parcellation. Each term in Fig. 7 denotes the major tasks involved in each cluster (with more details in Supplementary Figure S4). To evaluate the homogeneity of the 15 cluster parcellation, we calculated the DCBC. The strongest DCBC value was 0.10 at an equidistance of 40 mm (Fig. 3 right, Supplementary Table S2), demonstrating a positive homogeneity, which is lower than the DCBC of 0.16 for the clustering reported in King et al. [3]. We were not able to acquire the original clustering results of King et al. [3], so it is possible that these differences are also due to some implementational differences in computing the DCBC.

For convenience, Fig. 4 illustrates our reanalysis of King et al. [3] together with the other parcellations discussed earlier. There is good convergence between our parcellation and the reanalysis, with major networks at approximately the same locations, and much more so than the original analysis by King et al. [3]. In particular, in the reanalysis, we see again a *language* network on the left hemisphere that is even larger than in our parcellation, a *divided attention* network that is much smaller, a *limbic* network that is very small and mainly on the right hemisphere as in our parcellation, and an *executive control* and *mentalizing* network of a similar size. The *mentalizing* network consists of the left hemisphere largely of movies involving natural scenes and biological/animal activities. In this re-analysis, we kept the *action observation* network separate from the more overarching *sensorimotor* network, because this network is often neighboring the *mentalizing* network. This is likely the result of actively judging biological movement on its substantive (e.g., goal-related) or emotional (e.g., happy or sad) meaning, because recall was often required afterwards, even though the instruction was to simply watch the movement passively. Judging movement for its substantive or emotional meaning may often activate mentalizing areas in the posterior cerebellum (see meta-analysis by [18]). With respect to the *directed attention* network, it is possible that it rather reflects the *saccades* or *visual* network described in King et al. [3] and Ji et al. [2], respectively, given its location and underlying task (i.e., visual search).

A Rand index of 0.73–0.75 showed a high level of convergence of the reanalysis of King et al. [3] with prior task-free parcellations ([1, 2]; Table 2), with an equally high similarity for the individual networks (mean Rand = 0.76–0.78), which was roughly the same as the present 7-network parcellation (Supplementary Table S1). Before we end, it is important to note that the parcellation obtained in this reanalysis is constrained by seeking convergence with preexisting cerebellar parcellations and a post hoc subjective interpretation of the underlying task-related processes, unlike our parcellation, which was based on established and empirically derived descriptors [32]. This reanalysis nonetheless points to the possibility of convergent validity between parcellations based on distinct task sets.

## General Discussion

The aim of this analysis was to derive a comprehensive characterization of the functional structure of the human cerebellum. This was motivated by limitations of earlier functional parcellations of the cerebellum. Parcellations based on resting-state brain activation (e.g., [1, 2]) were limited because of the lack of evidence that these task-free results are indicative of the functionality of the major networks uncovered. A novel parcellation based on task-related brain activation [3] was limited in that the study was carried out on a limited number of participants and tasks, so that the results might have been biased and specific to tasks and sample. For instance, the functional topology left out many important high-level psychological functions such as social mentalizing (or theory of mind), while it did put an undue emphasis on other lower-level social processes such as action observation (or mirroring: [24, 49]). Moreover, the ontological descriptors of the underlying psychological processes were not very compelling because they were obtained post hoc, and differed from functional processes revealed by recent meta-analyses, perhaps most markedly for social cognition [18, 19, 25, 26]. In sum, earlier resting-state and task-based parcellations may have introduced a bias, leading to an underrepresentation of important functions such as language and social cognition, apart from motor, cognitive, and affective functions studied earlier in the human cerebellum.

### Parcellation Based on Mapping of Task-Related Conceptual Topical Terms

As an alternative strategy, we employed a very large existing database of task-related neuroimaging data, NeuroSynth [31], to create a parcellation that could comprehensively describe the functional organization of the cerebellum across a plethora of tasks and participants, so that potential biases in tasks and populations were minimized. Importantly, the NeuroSynth dataset goes together with functional descriptors that have been empirically validated and applied previously on this dataset [32]. NeuroSynth characterizes psychological functionality by topical key terms extracted from the articles in the database that reflect latent underlying mental functions associated with patterns of brain activity. These key terms therefore provide a strong empirical and compelling description of the psychological functions underlying brain activity. Based on these key descriptors, we selected 22 topics reflecting only functional processes, and eliminated topics related to specific anatomic brain areas or to psychological pathologies. For each topic, we selected from the 10 highest loading key terms in NeuroSynth, only those that were unique for a given topic, and as top descriptive term, we selected the term with the highest centrality (most often the term with the highest topic loading). This procedure protected as much as possible against contamination of other topical functions due to overlap in studies related to similar key terms.

The results generally show that our novel cerebellar parcellation does not outperform earlier task-related parcellations at a spatial level. Specifically, although cluster convergence with earlier task-free parcellations [1, 2] is roughly equivalent (cf. Rand index), the present parcellation is somewhat more fragmented and less spatially homogenous (cf. DCBC index; [3]). Note that this latter measure should be interpreted with some caution, because the spatial shape of boundaries between parcellations (irregular versus smooth curvatures; folding of the cortex) is not well controlled for. The novel contribution of our meta-analytic approach is that it is based on the functional terms of NeuroSynth and so provides a very valuable contribution for a functional labelling of psychological processes underlying the partitions of the cerebellum, thereby providing a much more thorough analysis of the task characteristics that activate cerebellar areas.

One could argue that our top-down topical approach using NeuroSynth is prone to biases arising from preconceived notions present in the published corpus. Topical key terms may reflect how the majority of researchers conceive of psychological key functions, but this is not necessarily an accurate reflection of fundamental neurological processes. No objective criterion exists to validate these psychological interpretations. Nevertheless, our labeling is less dependent on the preconceptions of individual authors from previous task-free and task-based parcellations, in contrast to the large (if perhaps biased) consensus accrued from most authors in the literature.

However, the choice of NeuroSynth as our database comes at a cost because, as intimated in the introduction, it has several potential limitations. We reiterate these here briefly. First, the database might be prone to a publication bias, collects a large variety of analytic parameters and choices, and provides only peak coordinates devoid of individual variability. Against this criticism, we argued, stands the sheer volume—over 40 000 participants involving the cerebellum—and variety of research designs, tasks, and topics, which allow for a generalization that may largely protect against these limitations, at least to the same extent as conventional meta-analyses in the literature. Second, the inferior cerebellum is incompletely covered or inappropriately analyzed in many studies [33]. Against this criticism stands the popular winner-takes-all approach in parcellation research, which compares activation at each individual voxel, and thus treats competitive parcellations and interpretation equally (although the data volume might be restricted). Finally, NeuroSynth is an automated database, often including irrelevant volume or connectivity analyses, inadequate coordinate conversions, or non-healthy participants. To avoid this limitation, all studies with cerebellar coordinates in the dataset were carefully screened one by one, so that only valid functional activations in MNI coordinates of healthy participants were included in the analysis. Nonetheless, it is important to take these limitations into account, to understand some of the shortcomings of our parcellation results.

### Our Task-Based Parcellation Converges with Earlier Resting-State Parcellations

Our analysis of the NeuroSynth database provided task-related functional clusters, which were very consistent with resting-state partitions published earlier, in particular Ji et al. [2] and Buckner [1]. We observed independent bilateral clusters representing *sensorimotor*, *divided (ventral) attention*, *directed (dorsal) attention*, *executive control*, and *mentalizing (default mode)* networks and a unilateral cluster reflecting a *language* network. Despite this high converge with prior well-known parcellations in the literature, this does not mean that our proposed network nomenclature corresponds exactly to prior proposed networks, or the functions associated with the NeuroSynth key terms. Therefore, some networks deserve additional clarifications.

The *divided (ventral) attention* network is closely related to the salience network [50] and cingulo-opercular network [51]. Seeley et al. [50] found empirical evidence for distinguishing the salience network encompassing the dorsal anterior cingulate (at the medial prefrontal cortex) from the *executive control* network comprising of the dorsolateral frontal and parietal cortices. They demonstrated that the salience network was functionally related to anxiety, and together with the inclusion of the dorsal anterior cingulate and its association with conflict monitoring [52, 53], this supports the present characterization of the *divided attention* network by the key terms “fear,” “inhibition,” and “conflict”. With respect to fear, an analysis of distinct emotional responses in relation to brain networks [54] seems to suggest that apart from the mentalizing (and visual) networks in the cerebrum, fear triggers most strongly the ventral attention network followed by the dorsal attention network. To our knowledge, no such in-depth analysis has been published on the relation of distinct emotions, such as fear, and networks in the cerebellum.

The *executive* network is covered by topical key terms that seem to reflect one of its basic components, namely updating. This executive process refers to updating and monitoring of working memory representations [55, 56] and has been linked to frontoparietal and medial temporal cortices [57]. It is reflected by our key terms “working memory” and “encoding,” and the dynamically manipulation of its predominantly cognitive (i.e., non-social) content in typical experimental tasks, reflected by our key terms “objects” and “number” as well as “prediction” and “events” (i.e., temporal sequencing). There is no evidence for a distinct representation of other executive components such as inhibition or shifting (i.e., switching; [55, 56]), although these component processes are likely incorporated under some tasks denoted by our key terms.

The *mentalizing* network is often denoted as *default mode* network because it is prominently active in task-free (hence the term *default mode*) circumstances, but it is becoming increasingly clear, also from this parcellation, that this network predominantly reflects *mentalizing* during social cognition, that is, understanding the mental state of self and other persons [58], also in the cerebellum [17,  26].

The *language* network is unilaterally located on the right hemisphere, which corresponds with its contralateral left-hemispheric dominant position in the neocortex. The role of the cerebellum in grammatical and verbal processes is well established [59]. Although the *language* network is spatially close to the *mentalizing* network in the present analysis, the identification of an independent language function is in line with recent findings suggesting that although there might be some overlap between language and mentalizing at the group level, these networks can be distinguished [2, 28, 60]. The spatial proximity is most likely because *language* is functionally related to *mentalizing* for at least two reasons. First, *language* and *mentalizing* involve relatively stimulus-independent processes without a tangible presence of objects or events, as *language* often refers to categories that require abstraction (e.g., a “bird” can be a sparrow, condor, pigeon, etc.; [61]) or refers to (past or future) states that do not exist at present (e.g., “tomorrow…”) just like *mentalizing* refers to non-perceptual mental states. Second, because *language* and especially narratives are a vehicle, whereby information is exchanged about human agents and their personal and social characteristics.

An independent cluster reflecting the *limbic* network was only minimally represented, much like other parcellations where it was not represented at all [2, 3]. This minimal representation is perhaps due to the fact that many emotional tasks focus on facial expressions, and perhaps therefore rather fell under the sensorimotor network in our analysis, or required an interpretation of sensorial states or emotional words [62], and so activated the mentalizing network. Recent meta-analyses confirm that implicit and explicit emotion tasks on self and others activate the cerebellar mentalizing network [18] as well as other cerebellar networks involving divided attention, sensorimotor activation and executive control, showing little evidence for a unique contribution of the limbic network [54, 63]. Another potential explanation is the normalization applied in our (and other) parcellations, which may have relatively disfavored emotion tasks (see Supplementary Figure S2).

Unlike the task-based parcellation by King et al. [3], we found no evidence for an independent cluster of *action observation*, which reflects a lower-level process of social cognition based on perception of biological movement (see meta-analyses by [24, 49]). Nor did we find evidence for an independent cluster of *autobiographic recall*, which is more typically conceived as a part of social mentalizing (see meta-analyses by [17, 18, 64]). It appears that these task-related clusters are not reliable or independent at the present level of analysis, and might perhaps be revealed as lower-level distinctions in a more fine-grained parcellation.

### Our Task-Based Parcellation Is Consistent

The cross-validation results showed a good split-half reliability of our parcellation. However, many voxels shifted their assignment after drawing random splits from the database. This is because assigning voxels to clusters depends on the winner-take-all approach, which may easily shift because of small differences in activation levels at the boundaries. This finding has important repercussion on the consistency of task-based cerebellar parcellations. It suggests that changes in data input from existent databases (e.g., NeuroSynth) or novel research (e.g., MDTB; [3]) are likely to change task-based parcellations significantly. One likely reason is that tasks lead to sharply delimited areas of activation, whereas resting state shows a much smoother pattern of varying activation across the whole brain. Another reason might be the limited quality of large databases like NeuroSynth, or the limited breadth of relevant tasks in novel research [3]. Changes in data input from tasks may therefore have larger effects than for resting state, rendering the latter parcellations more robust.

### Our Task-Based Parcellation Converges with a Reanalysis of an Earlier Functional Task Battery

Our analysis of the NeuroSynth database showed some convergence with another functional dataset originally used by King et al. [3], but now reanalyzed using procedures very similar to ours. Although the original and present (re)analyses are limited by a post hoc identification of clusters and networks, the comparison between our parcellation and the reanalysis of King et al. [3] demonstrates the possibility of convergent validity between parcellations based on distinct task and data sets. Although this convergence validates the same networks at roughly similar locations, convergence was limited at the fine-grained voxel level most probably for reasons of consistency with respect to task-based activation input explained in the previous paragraph.

### Asymmetries and Inhomogeneities in Our Task-Based Parcellation

A notable aspect of our parcellation and that of King et al. [3] is that these task-based parcellations were less symmetrical across the left and right cerebellar hemispheres than resting-state parcellations [1, 2]. In our parcellation, this hemispheric asymmetry was most evident with respect to the spatial volumes and location of clusters representing the *sensorimotor* and *divided attention* networks, which were sometimes very homogenous on one hemisphere and quite fragmented on the other. Other structures were more robust, homogenous, and extensive, such as the *executive control* and *mentalizing* networks. As mentioned before, our *limbic* cluster was minimally represented, in accord with King et al. [3].

The spatially disparate and asymmetric functional mappings of task-based versus resting-state parcellations suggests that finer details of the functional organization of the cerebellum are perhaps idiosyncratic for each individual and task. There are several reasons to support individual variation as explanation for the observed functional asymmetry. First, there are significant within-individual variations in the spatial location and extent of cerebellar mappings [34], which are claimed to be more variable than the cerebral cortex [65], as well as large gender differences in cerebellar volumes and connectivity [66, 67]. Second, neuroimaging research typically excludes individuals with left handedness, which may also have introduced a task-based asymmetry. Third, the NeuroSynth dataset includes many individuals participating in distinct tasks in different experiments, which increases variation even more. However, the fact that hemispheric asymmetry is revealed in both the present analysis (using different individuals) and King et al. [3] suggests that perhaps tasks rather than individuals contributed to this asymmetry. This coincides with the suggestion that tasks may range from engaging limited to multiple psychological subcomponents, recruiting brain areas ranging from functionally highly specialized to highly flexible [68]. The (non)participation of functionally specialized tasks may therefore have a substantial focal impact on a parcellation. In addition, procedural differences in the analyses (e.g., normalization and fMRI analysis versus ALE meta-analysis) may have further contributed to more differences and asymmetries in network localization and boundaries between the present task-based parcellation and the one from King et al. [3].

While all prior and the current parcellations provide evidence of critical functional distinctions underlying the organization of the human cerebellum, a general limitation is that these analyses have hereto not provided evidence on the process that distinguishes cerebellar from neocortical processes. Cross-validation with the neocortex in our analysis and in earlier work [1, 2] provides compelling evidence of the close connectivity between the cerebellum and the cerebral cortex. These tight links support the hypothesis that incoming cerebral signals undergo a transformational process in the cerebellum, which contributes to efficient and adaptive cortical processing and behavior [12, 69].

## Conclusion

This article describes a comprehensive functional parcellation for the human cerebellum, based on a cleaned-up large functional database, NeuroSynth, which is unique in its functional diversity of tasks and amount of data and participants, and the reliable association of tasks with functional key descriptors. The primary purpose was to describe the functions of the human cerebellum using descriptors of underlying psychological processes in a more reliable and compelling manner than earlier task-related parcellations. Our analysis revealed a partition that exhibited replicability with earlier task-free and task-related parcellations as well as between neocortical and cerebellar structures. We believe that the present parcellation presents a reasonably accurate estimate of cerebellar task-based functional network organization in humans so far, based on a large corpus of semantic terms characterizing researchers’ current consensus on psychological processes supported by the brain. Our functional organization confirms the major network at locations that are roughly similar to earlier resting-state parcellations [1, 3], including *sensorimotor*, *directed (dorsal) attention*, *divided (ventral) attention*, and *limbic* networks (but not the *visual* network) and shows strong convergence for higher-level associative networks involving *executive control*, *mentalizing (default mode)*, and *language* networks. However, like prior task-related partitions [3], the present results are less symmetrical across the two hemispheres. Given its extensive and solid ontological basis, for future research, the present parcellation and associated key terms can provide a more useful guide in designing studies to test specific functional hypotheses and provide a reference for interpreting the results.

### Supplementary Information

ESM 1

## Data Availability

Final cerebellum and MBDT parcellations, and program coding (MatLab scripts) are available via https://github.com/SocialCerebellum/Parcellation. Databases (Excel) and other files are available from the first author upon request.
