# Supplementary material for: A Functional Atlas of the Cerebellum Based on NeuroSynth Task Coordinates
Source: Cerebellum. 2023 Aug 22;23(3):993–1012. doi: 10.1007/s12311-023-01596-4 (PMC11102394; doi:10.1007/s12311-023-01596-4)
Supplement: Supplementary file 1 — ESM 1 [file 12311_2023_1596_MOESM1_ESM.docx]

**Supplementary Table S1**

| Table S1. (Adjusted) Rand index comparing the similarity in parcellations of task-free and task-based studies | | | | | | | | | | |
| --- | --- | --- | --- | --- | --- | --- | --- | --- | --- | --- |
|  |  | Rand index | | | |  | Adjusted Rand index | | | |
|  |  | 1 | 2 | 3 | 4 |  | 1 | 2 | 3 | 4 |
|  |  | All Networks | | | | | | | | |
| 1 | Buckner et al. (2011) |  |  |  |  |  |  |  |  |  |
| 2 | Ji et al. (2019) | 0.78 |  |  |  |  | 0.27 |  |  |  |
| 3 | King et al. (2019) | 0.75 | 0.75 |  |  |  | 0.23 | 0.19 |  |  |
| 4 | Reanalysis of King et al. (2019) | 0.75 | 0.73 | 0.75 |  |  | 0.22 | 0.14 | 0.26 |  |
| 5 | Present 7-network parcellation | 0.67 | 0.66 | 0.65 | 0.65 |  | 0.11 | 0.07 | 0.08 | 0.07 |
|  |  | Means of all individual Networks | | | | | | | | |
| 2 | Ji et al. (2019) | 0.80 |  |  |  |  | 0.25 |  |  |  |
| 3 | King et al. (2019) | 0.72 | 0.73 |  |  |  | 0.06 | 0.19 |  |  |
| 4 | Reanalysis of King et al. (2019) | 0.75 | 0.76 | 0.76 |  |  | 0.13 | 0.13 | 0.09 |  |
| 5 | Present 7-network parcellation | 0.74 | 0.75 | 0.73 | 0.73 |  | 0.10 | 0.10 | 0.02 | 0.07 |
|  |  | Sensorimotor Network | | | | | | | | |
| 2 | Ji et al. (2019) | 0.74 |  |  |  |  | 0.38 |  |  |  |
| 3 | King et al. (2019) | 0.73 | 0.69 |  |  |  | 0.41 | 0.28 |  |  |
| 4 | Reanalysis of King et al. (2019) | 0.68 | 0.66 | 0.73 |  |  | 0.33 | 0.26 | 0.44 |  |
| 5 | Present 7-network parcellation | 0.67 | 0.72 | 0.64 | 0.60 |  | 0.15 | 0.16 | 0.13 | 0.11 |
|  |  | Directed (Dorsal) Attention Network | | | | | | | | |
| 2 | Ji et al. (2019) | 0.74 |  |  |  |  | 0.23 |  |  |  |
| 3 | King et al. (2019) | 0.84 | 0.74 |  |  |  | 0.00 | 0.00 |  |  |
| 4 | Reanalysis of King et al. (2019) | 0.79 | 0.69 | 0.92 |  |  | 0.02 | -0.04 | 0.00 |  |
| 5 | Present 7-network parcellation | 0.81 | 0.72 | 0.96 | 0.89 |  | -0.03 | -0.02 | 0.00 | -0.02 |
|  |  | Divided (Ventral) Attention Network | | | | | | | | |
| 2 | Ji et al. (2019) | 0.72 |  |  |  |  | 0.27 |  |  |  |
| 3 | King et al. (2019) | 0.52 | 0.57 |  |  |  | -0.02 | 0.05 |  |  |
| 4 | Reanalysis of King et al. (2019) | 0.65 | 0.75 | 0.62 |  |  | 0.03 | 0.11 | 0.14 |  |
| 5 | Present 7-network parcellation | 0.55 | 0.53 | 0.50 | 0.52 |  | 0.07 | 0.01 | -0.01 | -0.02 |
|  |  | Limbic Network | | | | | | | | |
| 2 | Ji et al. (2019) | 0.89 |  |  |  |  | 0.00 |  |  |  |
| 3 | King et al. (2019) | 0.89 | 1.00 |  |  |  | 0.00 | 1.00 |  |  |
| 4 | Reanalysis of King et al. (2019) | 0.89 | 0.99 | 0.99 |  |  | -0.01 | 0.00 | 0.00 |  |
| 5 | Present 7-network parcellation | 0.89 | 1.00 | 1.00 | 0.99 |  | 0.00 | 0.00 | 0.00 | 0.00 |
|  |  | Executive Control Network | | | | | | | | |
| 2 | Ji et al. (2019) | 0.68 |  |  |  |  | 0.33 |  |  |  |
| 3 | King et al. (2019) | 0.65 | 0.59 |  |  |  | 0.00 | 0.00 |  |  |
| 4 | Reanalysis of King et al. (2019) | 0.62 | 0.59 | 0.61 |  |  | 0.19 | 0.14 | 0.00 |  |
| 5 | Present 7-network parcellation | 0.55 | 0.54 | 0.54 | 0.51 |  | 0.09 | 0.07 | 0.00 | 0.01 |
|  |  | Mentalizing (Default Mode) Network | | | | | | | | |
| 2 | Ji et al. (2019) | 0.81 |  |  |  |  | 0.53 |  |  |  |
| 3 | King et al. (2019) | 0.67 | 0.77 |  |  |  | 0.00 | 0.00 |  |  |
| 4 | Reanalysis of King et al. (2019) | 0.74 | 0.77 | 0.70 |  |  | 0.39 | 0.41 | 0.00 |  |
| 5 | Present 7-network parcellation | 0.74 | 0.78 | 0.73 | 0.72 |  | 0.39 | 0.42 | 0.00 | 0.31 |
|  |  | Language Network | | | | | | | | |
| 2 | Ji et al. (2019) | 0.99 |  |  |  |  | 0.00 |  |  |  |
| 3 | King et al. (2019) | 0.73 | 0.73 |  |  |  | 0.00 | 0.00 |  |  |
| 4 | Reanalysis of King et al. (2019) | 0.91 | 0.91 | 0.71 |  |  | 0.00 | 0.05 | 0.07 |  |
| 5 | Present 7-network parcellation | 0.98 | 0.97 | 0.72 | 0.91 |  | 0.00 | 0.07 | 0.00 | 0.11 |
| *Note*: This analysis is based on the 7-networks of Buckner et al. (2011) and an additional language network of Ji et al. (2019), and uses parcellations based on merged clusters in studies 4 - 5. All other clusters were collapsed in an 'other' category. For individual network analyses, the networks not involved were also assigned to a single 'other' category'. | | | | | | | | | | |

**Supplementary Table S2**

| Table S2: Correlations for ‘Within’ and ‘Between’ voxel pairs and resulting DCBC difference for the present 7-network parcellation and reanalysis of King et al. (2019) | | | | | | | | | | | |
| --- | --- | --- | --- | --- | --- | --- | --- | --- | --- | --- | --- |
|  | Present 7-network parcellation | | |  | Present 10-cluster parcellation | | |  | Reanalysis of King et al. (2019) | | |
| Spatial distance (mm) | Within | Between | DCBC |  | Within | Between | DCBC |  | Within | Between | DCBC |
| 6 | 0.75 | 0.75 | 0.00 |  | 0.75 | 0.75 | 0.00 |  | 0.75 | 0.75 | 0.00 |
| 10 | 0.51 | 0.49 | 0.02 |  | 0.51 | 0.49 | 0.02 |  | 0.51 | 0.49 | 0.02 |
| 16 | 0.30 | 0.26 | 0.04 |  | 0.31 | 0.26 | 0.05 |  | 0.31 | 0.26 | 0.05 |
| 20 | 0.17 | 0.12 | 0.05 |  | 0.18 | 0.12 | 0.06 |  | 0.18 | 0.13 | 0.06 |
| 26 | 0.10 | 0.03 | 0.07 |  | 0.11 | 0.03 | 0.08 |  | 0.12 | 0.04 | 0.08 |
| 30 | 0.05 | -0.04 | 0.08 |  | 0.06 | -0.03 | 0.09 |  | 0.05 | -0.03 | 0.08 |
| 36 | 0.03 | -0.06 | 0.09 |  | 0.04 | -0.06 | 0.09 |  | 0.03 | -0.05 | 0.08 |
| 40 | 0.04 | -0.07 | 0.10 |  | 0.04 | -0.06 | 0.10 |  | 0.04 | -0.05 | 0.10 |
| 46 | 0.04 | -0.05 | 0.09 |  | 0.03 | -0.04 | 0.07 |  | 0.03 | -0.04 | 0.06 |
| 50 | 0.00 | -0.08 | 0.07 |  | -0.01 | -0.07 | 0.07 |  | 0.01 | -0.07 | 0.08 |
| *Note*: All correlations are significant at *p* < .00001 | | | | | | | | | | | |

**Supplementary Table S3**

| Table S3: Mean Rand values across 20 repetitions | | |
| --- | --- | --- |
| Network | Rand | Adjusted Rand |
| Mentalizing | 0.76 | 0.34 |
| Divided attention | 0.70 | 0.18 |
| Executive | 0.73 | 0.24 |
| Limbic | 0.87 | 0.68 |
| Directed attention | 0.86 | 0.64 |
| Language | 0.88 | 0.71 |
| Sensorimotor | 0.85 | 0.60 |
| Mean | 0.81 | 0.48 |

**Supplementary Figure S1**: Activation on cerebellar flatmaps for all individual NeuroSynth topics. Shown are ALE z-values for the selected 22 NeuroSynth topics, with their NeuroSynth topic number. Warmer colors indicate higher activation. The numbers in the legend refer to the cerebellar lobules, with lobule VIIa split up as Crus 1 and 2 (Diedrichsen & Zotow, 2015).

**Supplementary Figure S2:** Parcellation of the cerebellum into 10 functional clusters, with or without prior normalization of the ALE z-scores and displayed on a cerebellar flatmap, smoothed with a 5 isometric voxels box filter. The normalized parcellation is most similar to the other resting-state parcellations and re-analysis of King et al. (2019; see Figure 4). Although clusters tend to occupy roughly the same locations, there are large differences in the spatial extent of the clusters given the two normalization approaches. Clear differences in the non-normalized parcellation are, for instance, the much larger Sensorimotor (1) and Directed Attention networks (2), and the much smaller Divided Attention network (3); the Mentalizing network (10) which is largely "covered” by much larger Language (4) and Limbic clusters (5 & 6). This seems to indicate that stronger raw ALE z-scores in the non-normalized parcellation (e.g., Sensorimotor, Directed Attention, Language, and Limbic clusters) became relatively weaker after normalization.

**Supplementary Figure S3**: Activation on the cerebral cortex of all individual NeuroSynth topics. Shown are whole-brain ALE z-values for the 22 NeuroSynth topics, combined per (sub)cluster shown on a medial section and left hemisphere of the Colin cortex using Mango. Mean titles refer to networks, with underneath the NeuroSynth topics belonging to the network. First topic = 1^st^ red layer, second topic = 2^nd^ green layer, third topic = 3^th^ blue layer; layers may overlap. Networks with more than 3 topics are shown by 2 or 3 groupings of topics. The Yeo atlas on the right refers to Figure 6 (right panel), and is shown for reference. Note that the visual network was not present in the cerebellar parcellation.

**Supplementary Figure S4**: Functional cluster analysis on the brain activations of the 61 tasks in the multidimensional task battery (MDTB) of King et al. (2019). The vertical broken line demarcates the solution for 15 clusters. The bar on the right characterizes each cluster by the most dominant tasks, and their most likely assignment to major networks consistent with the present parcellation, using the color coding of Figure 7 (MDTB parcellation).

Supplementary Figure S1


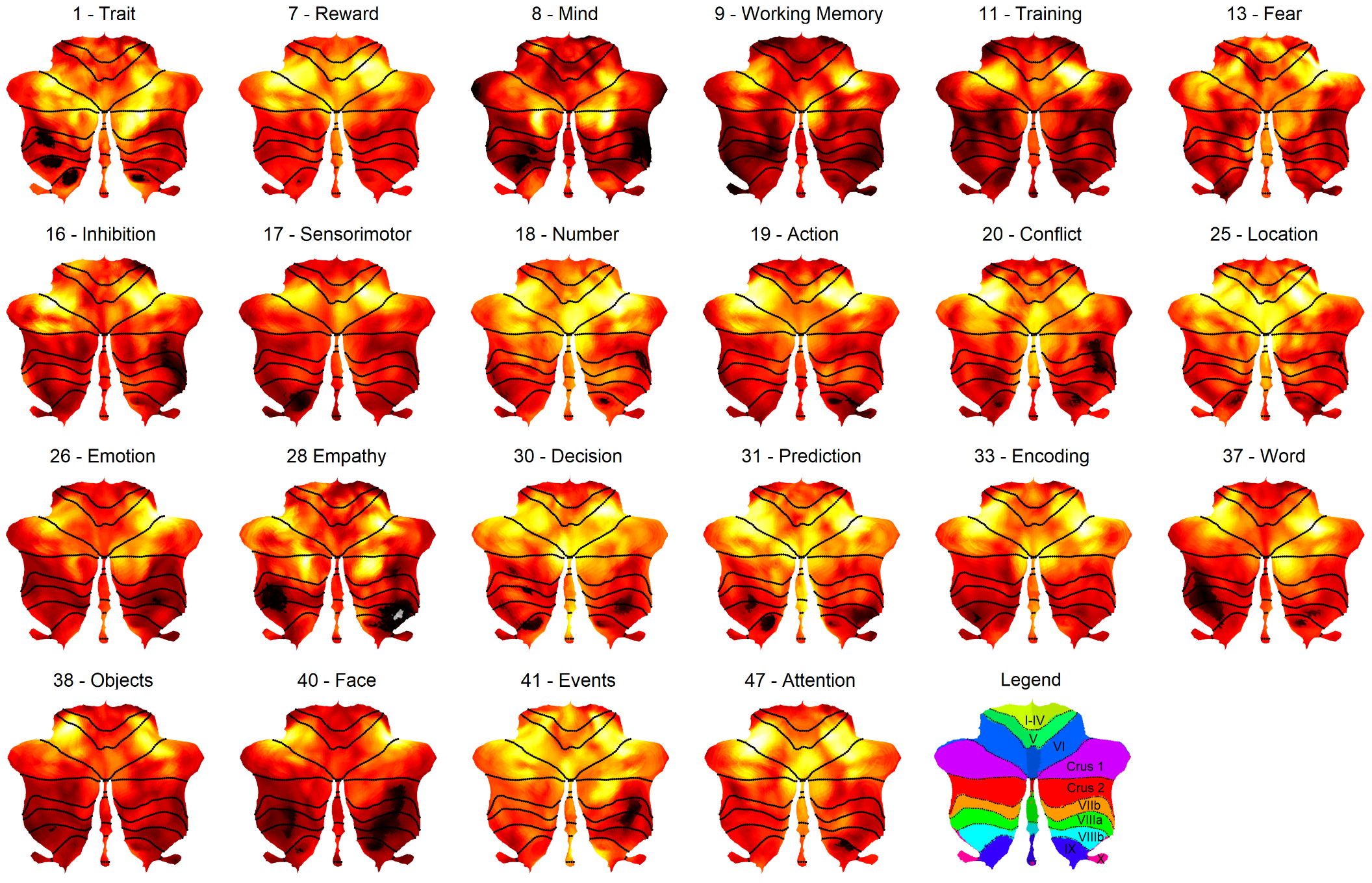


Supplementary Figure S2


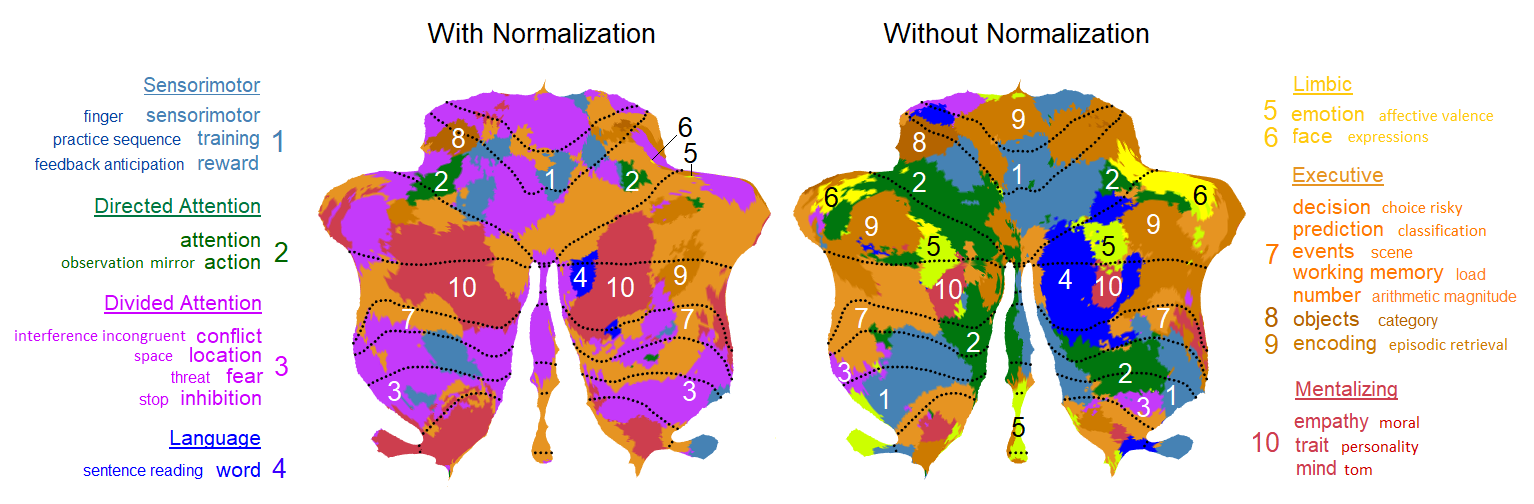
Supplementary Figure S3


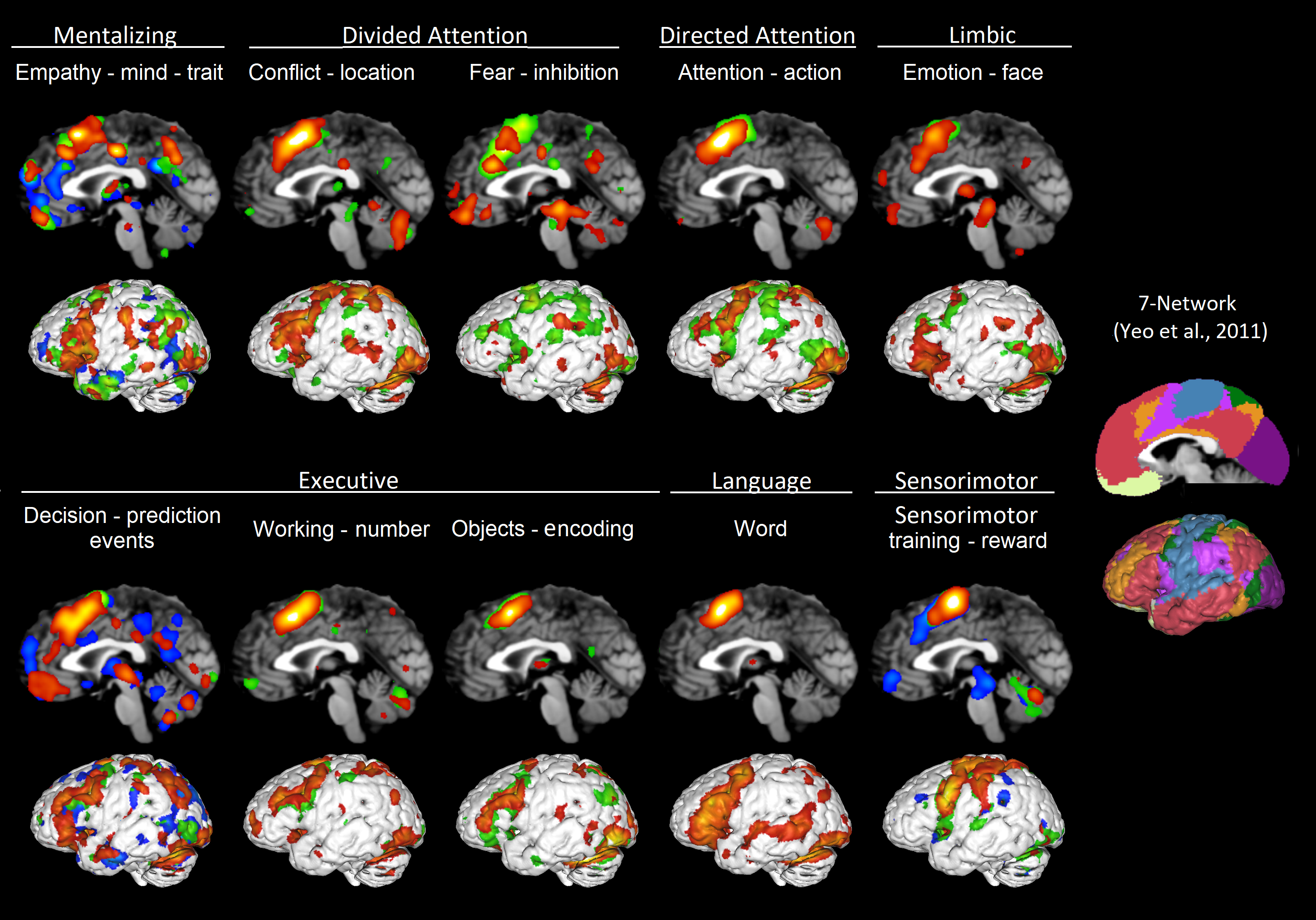


Supplementary Figure S4

###
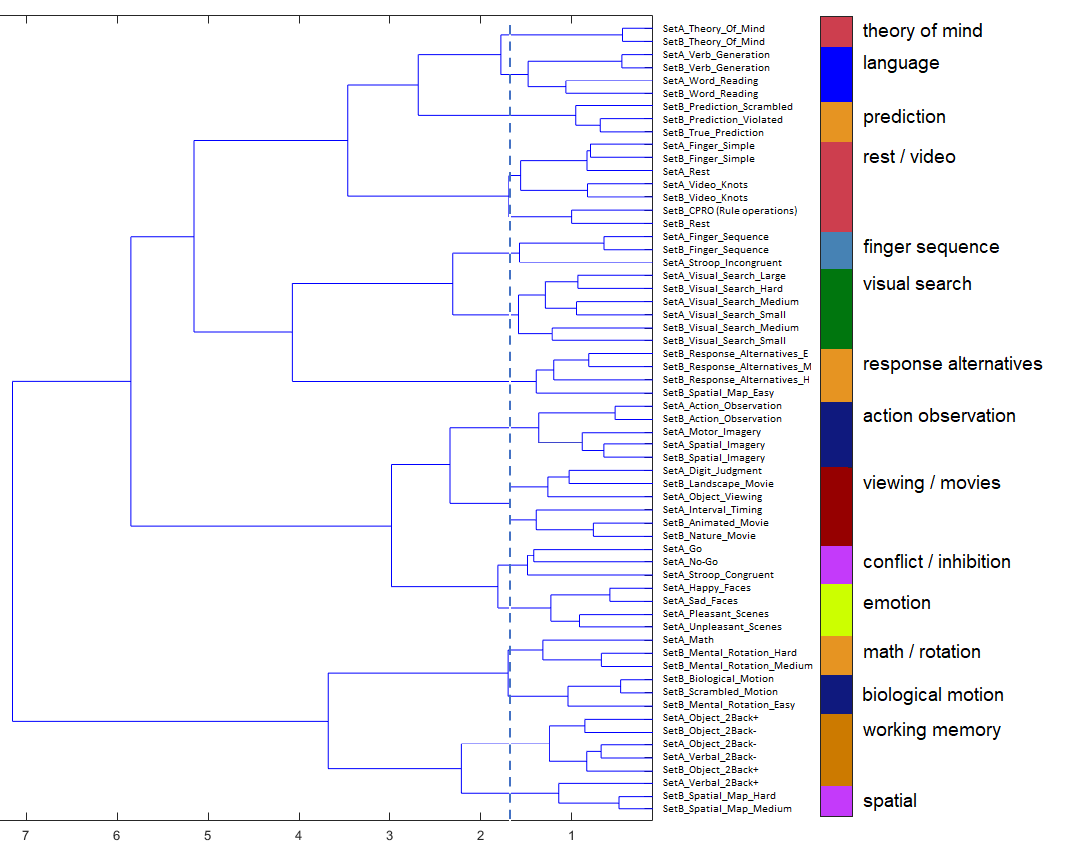


### *Files related to the distinct step in the analysis*

***Selection of Cerebellar Coordinates***

1. Accessing NeuroSynth Database on 12 November 2020:

saved in Excel file database_select

1. Selection of a cerebellum mask (ROI) by anatomical boundaries

- Within a box defined by the outer x-y-z coordinates -63 > x < 63; -28 > y >-103; and z < 11

(Excel database_CE_ROI.xlsx in columns “box_x, box_y, box_z”)

- Below the z coordinates surrounding the superior part of the cerebellum determined by two planes defined by x-y-z coordinates: 0 -50 12 and ±60 -50 -30, using the formulas: z < (-30 -12) / (60 -0) * ABS(x) +12; and z < -0.70 * ABS(x) +12

(Excel database_CE_ROI.xlsx in column “0 -50 12 / 60 -50 -30 plane”)

- Below the z coordinates surrounding the declining posterior part of the cerebellum determined by x-y-z coordinates: 0 -50 12 and 0 -100 -15

(Excel database_CE_ROI.xlsx in column “0 -50 12 / 0 -100 -15 plane”)

1. Selection using Brain Atlases:

- Using the SPM Anatomy box

Excel database_CE_ROI.xlsx in columns following “**SPM ANATOMY”**

(resulting in n = 26601 coordinates; n = 5726 articles

- Using the Talairach Client toolbox

Excel database_CE_ROI.xlsx in columns following “**TALAIRACH CLIENT**”

resulting in 29470 coordinates; 6177 articles

1. Selection of only MNI coordinates:

Excel database_CE_pubs_tabs_MNI to be validated by the Dwarfs

resulting in 4716 articles with MNI coordinates

***Selection of Functional NeuroSynth Topics and Terms***

1. Extracting Topics from NeuroSynth website

https://neurosynth.org/analyses/topics/v5-topics-50/

saved in Excel file v5-topics-50.xlsx

1. Selecting top 10 terms for each NeuroSynth topic

Excel v5-topics-50.xlsx columns following “Top 10 terms”

1. Excluding non-functional terms

Excel v5-topics-50.xlsx column “Selected” switched to “0”

1. Selecting only unique terms among the remaining functional terms

Excel v5-topics-50.xlsx columns “Uniqueness” parameter as low as possible and close to 1 and the columns under the heading “Unique terms top 10”

1. Final selection of representative functional terms

computed for overlap in Excel database_pubs_overlap_000_term and for uniqueness in Excel database_pubs_centrality_000_term.xlsx

***Quality screening of Selected Studies and Coordinate Tables***

1. Excluding studies or tables referring to analyses with (see method section)
2. Including studies or tables referring to analyses with (see method section)
3. Combining all databases from individual collaborators (i.e., dwarfs)

database_CE_pubs_tabs_MNI_NOterms_AllDwarfs

database_CE_pubs_tabs_MNI_NOterms_AllDwarfs_ValidityCheck

database_CE_pubs_tabs_MNI_NOterms_AllDwarfs_ValidityCheck_Sorted

database_CE_pubs_tabs_MNI_NOterms_AllDwarfs_Valid

database_CE_pubs_tabs_Valid for all validated studies and tables

1. Adding number of Participants:

database_CE_pubs_tabs_MNI_NOterms_Valid with 50325 participants

1. Adding coordinates

Excel database_CE_valid validated for all 1873 studies and 12485 coordinates taken from the whole CE selected database

***Functional Parcellation***

1. Preparing input for ALE Analysis of functional topics

MatLab: run_dwarfs_ALEinput

1. ALE Analysis of individual topics

GingerAle run manually using the graphic interface

1. Clustering of functional topics

MatLab: run_dwarfs_cluster

Note: uses the standard MatLab function “clusterdata”

1. Assigning clusters to anatomical areas using a winner-take-all principle

MatLab: run_dwarfs_winner

1. Stability of functional parcellations

MatLab: run_dwarfs_DCBC

1. Similarity of functional parcellations

MatLab: run_dwarfs_rand

Note: uses the publicly available rand MatLab function from:

https://nl.mathworks.com/matlabcentral/fileexchange/49908-adjusted-rand-index

1. Cross-validation: split-half reliability (ALE analyses of random halves of the data)

MatLab: run_dwarfs_validate

1. Cross-validation: split-half reliability (Pearson and Rand indices of winning clusters)

MatLab: run_dwarfs_val_winner

Note: uses the same MatLab functions described above
